# Hierarchical Indexing with Controlled Expansion for Efficient Semantic Search over Encrypted Cloud Data

**DOI:** 10.3390/e28070721

**Published:** 2026-06-24

**Authors:** Yu Zhang, Rui Zhu, Yin Li

**Affiliations:** 1 School of Computer and Information Technology, Xinyang Normal University, Xinyang 464000, China; zhangyu86@xynu.edu.cn (Y.Z.); zhurui@xynu.edu.cn (R.Z.); 2 School of Cyberspace Security, Dongguan University of Technology, Dongguan 523000, China

**Keywords:** searchable encryption, semantic retrieval, hierarchical index, query expansion, privacy preservation

## Abstract

The proliferation of cloud-based data outsourcing has intensified the need for efficient semantic retrieval over encrypted data. Existing searchable encryption schemes often face a coupled bottleneck: (i) semantic index can be unstable or overly coarse, yielding loose pruning bounds and high query cost, and (ii) semantic query expansion can easily introduce noise, forcing an unfavorable accuracy–efficiency trade-off. To address these issues, we propose SES-HI, a Semantically Enhanced Searchable Encryption scheme with a stability-oriented hierarchical index for efficient ranked semantic search over encrypted cloud data. SES-HI contains three core innovations. First, it constructs a balanced ω-ary hierarchical index using a two-stage clustering pipeline (Ward → k-means) to produce semantically compact groups and more representative node vectors, enabling tighter pruning bounds. Second, it performs topic-guided query expansion using LDA and applies Word2Vec-based similarity filtering to enrich semantic coverage while suppressing expansion noise. Third, it introduces a dual-pruning strategy that couples a global threshold with top-*k* competitive pruning to reduce traversal and ranking overhead without sacrificing recall. We formally prove that SES-HI is secure against adaptive chosen-keyword attacks under an explicit leakage profile. Extensive experiments on the TREC dataset demonstrate that SES-HI consistently improves the accuracy–latency trade-off compared with state-of-the-art baselines, supporting practical semantic search for privacy-sensitive cloud applications.

## 1. Introduction

Cloud computing has become the default platform for storing and analyzing large-scale textual data. To protect confidentiality, data owners routinely encrypt documents before outsourcing them to the cloud. Encryption, however, breaks the ability of the cloud server to interpret and respond to users’ information needs, creating a fundamental tension between privacy and usability. Searchable Encryption (SE) [1] addresses this tension by enabling servers to filter encrypted data without decrypting it. As real-world demands evolve from exact matching to intent-driven search, SE research has increasingly moved toward ciphertext retrieval that supports semantic understanding and relevance ranking.

Early SE systems [2,3,4] primarily focused on exact keyword matching. While efficient, exact matching cannot capture semantic relatedness between a user’s intent and document content. Semantic-aware ranked retrieval typically adopts a vector space model in which queries and documents are embedded into vectors and compared using similarity measures (e.g., inner product). Early semantic approaches [5,6] mainly relied on statistical features such as TF-IDF, which are vulnerable to vocabulary mismatch and have limited ability to represent contextual semantics. More recently, topic models (e.g., LDA) [7] and word embeddings (e.g., Word2Vec) [8] have been introduced to strengthen semantic representations. Representative directions include combining topic vectors with tree-based indexes to balance precision and efficiency [9], or organizing documents through clustering and using embedding-driven query expansion to improve relevance [10]. Despite progress, existing schemes still struggle to deliver a robust accuracy–efficiency balance in large-scale encrypted retrieval.

The difficulty in achieving this balance arises from a twofold bottleneck. (i) Unstable semantic index undermines pruning. In hierarchical encrypted search, the vectors at internal nodes must accurately summarize the semantic scope of their subtrees to enable effective pruning. However, due to clustering instability or coarse aggregation methods, these vectors can become unrepresentative. This results in loose upper bounds, severely degrading pruning efficiency. (ii) The noise–recall dilemma in query expansion. While semantic expansion is necessary to bridge the vocabulary gap between queries and documents, naive methods introduce semantically drifting terms. This creates a fundamental trade-off: schemes that prioritize speed through aggressive pruning may sacrifice recall by suppressing true positives, whereas those designed to preserve recall often incur high efficiency costs.

To address these coupled challenges, we propose SES-HI (Semantically Enhanced Searchable Encryption with Hierarchical Index), aiming to achieve a more optimal balance between retrieval accuracy and query efficiency in cloud environments. The key idea is to (i) stabilize and tighten the pruning upper bounds by constructing semantically compact clusters and a balanced hierarchical index, and (ii) control expansion noise through topic guidance and embedding-based filtering, while (iii) accelerating retrieval using a dual-pruning execution strategy that explicitly leverages the index bounds.

Contributions. The core contributions of this paper are threefold:

- Stability-Oriented Hierarchical Index with Tighter Pruning Bounds. We design an ω-ary balanced search tree driven by a two-stage clustering pipeline (Ward → k-means) to form semantically compact clusters and more representative internal-node vectors. This design directly targets the pruning degradation caused by unstable or overly coarse semantic summaries, and it provides the structural basis for fast traversal at scale.
- Topic-Guided, Embedding-Filtered Query Expansion for Noise Control. We propose a semantic query processing method that uses LDA to guide expansion toward plausible topical neighborhoods and applies Word2Vec similarity filtering to suppress semantic drift. This component is essential to improve semantic coverage without inflating the candidate set with low-relevance terms.
- Dual Pruning Mechanism for Practical Top-*k* Retrieval. We introduce a dual-pruning mechanism that combines a global threshold (for coarse elimination) with top-*k* competitive pruning (for fine-grained candidate maintenance). Their combination significantly enhances overall retrieval efficiency while ensuring high recall.

We provide a systematic privacy-leakage analysis of SES-HI, formally define its leakage function, and prove security under indistinguishability against adaptive chosen-keyword attacks (IND2-CKA) [2,3] within this leakage framework. Comprehensive experiments on the standard TREC dataset demonstrate that SES-HI outperforms mainstream baselines in both retrieval quality and query latency, validating its effectiveness in balancing semantic accuracy and encrypted search efficiency.

Organization. The remainder of this paper is structured as follows. Section 2 reviews related work. Section 3 elaborates the system model and security definition. Section 4 introduces necessary techniques in the proposed scheme. Section 5 presents the index construction and search methods in plaintext domain. Section 6 details the SES-HI scheme. Section 7 provides a formal security analysis. Section 8 presents the theoretical complexity analysis and experimental results. Finally, Section 9 concludes the paper and outlines future work.

## 2. Related Work

Searchable encryption has evolved from exact keyword matching to ranked, semantic, and structure-aware retrieval over encrypted data. Existing studies can be summarized from four directions, namely exact keyword searchable encryption, query expansion, semantic searchable encryption, and efficient index structures. This section briefly reviews these works and clarifies the relationship between SES-HI and representative schemes.

Searchable Encryption Schemes Supporting Exact Keyword Matching. Early searchable encryption mainly focused on exact keyword matching. Song et al. introduced a practical symmetric searchable encryption framework for encrypted keyword search [1]. Goh et al. and Curtmola et al. further proposed secure indexes and formal leakage-aware SSE definitions [2,3]. Cash et al. extended SSE to large-scale Boolean queries [4]. Later works considered multi-user authorization, multi-keyword ranked search, dynamic updates, similarity-based ranking, attribute-based search, Boolean search, and public-key searchable encryption [11,12,13,14,15,16,17,18]. DPMRS is a recent multi-keyword ranked search scheme that reduces high-dimensional TF-IDF cost through dictionary partitioning and multi-level indexing [19]. However, it is still mainly lexical-feature-driven and does not focus on controlled semantic expansion or pruning-aware hierarchical semantic retrieval.

Searchable Encryption Schemes Supporting Query Expansion. To overcome vocabulary mismatch, query expansion has been introduced into encrypted retrieval. Early studies used synonym dictionaries, association rules, and statistical co-occurrence information to enrich encrypted queries [20,21]. Fu et al. and Dai et al. further explored semantic-aware encrypted search and semantic-aware multi-keyword ranked retrieval [22,23]. CASE-SSE is a representative scheme in this direction. It uses Word2Vec-related semantic extension to improve encrypted search over outsourced data [10]. However, insufficiently controlled expansion can introduce weakly related terms and cause semantic drift. Although pre-trained language models and query-prediction methods have been studied in plaintext retrieval [24,25], directly applying them to encrypted retrieval often increases computation and system overhead. Therefore, lightweight and controllable query expansion remains necessary.

Searchable Encryption Schemes Supporting Semantic Search. Semantic searchable encryption represents documents and queries as semantic vectors rather than only keyword sets. Early ranked schemes mainly relied on bag-of-words or TF-IDF-style vectors [5,26]. Later works introduced Word2Vec and LDA to capture word-level associations and latent topic semantics [27,28]. EPSMR combines LDA-based topic vectors with a multi-branch tree index to improve privacy-preserving semantic ranked retrieval [9]. However, its query side does not include the controlled LDA and Word2Vec joint expansion mechanism used in SES-HI. Compass improves encrypted semantic search accuracy with pre-trained models, but its ORAM-based protection introduces relatively high overhead [29]. PBINS organizes semantic document vectors into K-means-based private bins and supports retrieval through range-encoded bitmap indexes and encrypted bitmap access [30]. It can benefit from SBERT or Transformer-style embeddings, but its core design is private-bin organization and bitmap-based access rather than hierarchical pruning. SSS KG enhances semantic association through knowledge graph modeling and encrypted graph search [31]. It can express richer entity-relation semantics, but its effectiveness depends on entity extraction, relation extraction, query graph construction, and graph matching quality.

Evolution of Index Structures. Efficient index design is essential for practical encrypted retrieval. Linear scanning was gradually replaced by secure indexes, inverted indexes, and tree-based indexes [2,3,6,32]. Xia et al. constructed a pruned balanced tree for secure dynamic multi-keyword ranked search [6]. EPSMR adopts a multi-branch tree aligned with topic vectors [9]. DPMRS uses dictionary-partition-based and double-tier indexes to reduce storage and search costs [19]. PBINS follows another route by combining K-means private bins with range-encoded bitmap indexes [30]. These studies show that index organization directly affects search cost, storage overhead, and retrieval quality. However, many existing tree-based or bin-based structures do not explicitly optimize semantic compactness inside indexed groups. Ward clustering and K-means are classical clustering methods [33,34], but using either one alone may suffer from high computational cost or sensitivity to initialization. Therefore, jointly considering semantic compactness, hierarchical organization, and pruning behavior remains important for encrypted semantic retrieval.

Relationship to Existing Works. SES-HI is related to EPSMR, CASE-SSE, PBINS, SSS KG, and DPMRS, but differs in its joint design objective. Like EPSMR, SES-HI uses topic vectors and tree-style indexing, but further builds a semantically compact ω-ary balanced tree through Ward followed by K-means and combines global threshold pruning with top-*K* competitive pruning. Like CASE-SSE, SES-HI exploits Word2Vec-related semantics, but uses LDA topic guidance and Word2Vec similarity filtering to control expansion noise. PBINS benefits from private bins and Transformer-style sentence embeddings, whereas SES-HI emphasizes lightweight topic-space representation and hierarchical pruning. SSS KG relies on knowledge graph construction and graph matching, while SES-HI avoids entity-relation extraction and better fits ordinary text collections. DPMRS reduces high-dimensional lexical-vector cost, whereas SES-HI targets semantic searchable encryption by jointly considering representation, expansion, indexing, and pruning. Therefore, SES-HI contributes an encryption-compatible framework for improving the accuracy–efficiency trade-off in encrypted semantic search.

## 3. System Model and Security Definition

This section formalizes the framework for our scheme. We define the system model with its entities and algorithms. The information exposed to the server is captured through explicit leakage functions. Based on this, we present the security definition associated with the proposed scheme.

### 3.1. System Model

**Figure 1:**
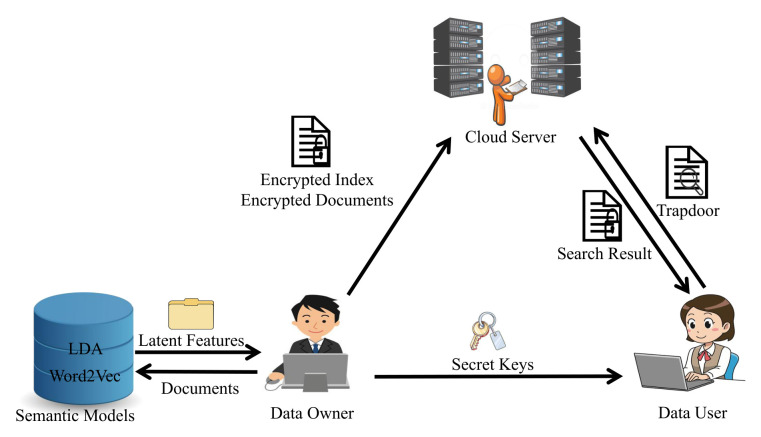
System architecture and workflow of the proposed scheme.

This section presents the generic system model of the proposed scheme. As illustrated in Figure 1, the model comprises three core entities: the Data Owner, the Cloud Server, and the Data User. The roles and responsibilities of these entities are defined as follows:

- Data Owner (DO): Serves as the trusted authority that generates the system’s secret key during initialization. The DO preprocesses its collection of plaintext documents and, using the secret key, locally executes the index construction algorithm (IndexBuild) to generate a searchable encrypted index. The encrypted index along with the encrypted documents are then outsourced to the cloud server.
- Cloud Server (CS): Provides massive storage and computational resources. The CS stores the encrypted indexes and document ciphertexts uploaded by DO. Upon receiving a query trapdoor generated by a Data User, the CS executes the encrypted search algorithm (Search) without decryption, returning the identifiers of the top-*K* most relevant documents.
- Data User (DU): An authorized entity that performs similarity search. The DU obtains the necessary secret key from the DO through a secure channel. Using this key, the DU transforms a query request into a secure query trapdoor. Furthermore, the DU decrypts and processes the encrypted search results returned by the CS to recover the required plaintext information.

Based on the above roles, the proposed scheme is formally defined by the following four polynomial-time algorithms:

- $\mathrm{Setup}(1^{\lambda })\to sk$: The initialization algorithm, executed by the DO. On input of a security parameter λ, it outputs a system secret key sk, which is then securely distributed to all authorized DUs.
- IndexBuild(sk,D)→I: The index generation algorithm, executed by the DO. It takes the secret key sk and a plaintext document set $D={\left\{\left({\mathrm{fid}}_{i},d_{i}\right)\right\}}_{i=1}^{n}$ as input, where ${\mathrm{fid}}_{i}$ is a document identifier and $d_{i}$ is the corresponding plaintext document. The algorithm outputs a structured encrypted index I.
- $\mathrm{TrapdoorGen}\left(sk,Q\right)\to T_{Q}$: The trapdoor generation algorithm, executed by the DU. Given the secret key sk and a query request *Q*, it outputs a secure query trapdoor $T_{Q}$. This trapdoor enables the server to perform the similarity search over the encrypted index without revealing any information.
- $\mathrm{Search}\left(I,T_{Q},K\right)\to {\mathrm{Res}}_{K}$: The secure retrieval algorithm, executed by the CS. Upon receiving the encrypted index I, a query trapdoor $T_{Q}$, and a parameter *K*, the server performs similarity search in the ciphertext domain and returns the set of top-*K* document identifiers ${\mathrm{Res}}_{K}=\left\{{\mathrm{fid}}_{1},\ldots ,{\mathrm{fid}}_{K}\right\}$ along with their associated encrypted documents.

Correctness Definition. The correctness of the scheme requires that for any legitimately generated key and index, and for any legitimate query, the Search algorithm returns exactly the same top-*K* document set as would be obtained by performing the same retrieval operation in the plaintext domain. Formally, this is defined as follows:

Consider a secret key $sk\leftarrow \mathrm{Setup}(1^{\lambda })$ generated from a security parameter λ, and an arbitrary document set D consisting of *n* documents. Let I←IndexBuild(sk,D) be the corresponding encrypted index. For any query request *Q*, let $T_{Q}\leftarrow \mathrm{TrapdoorGen}\left(sk,Q\right)$ be its query trapdoor. Define a plaintext retrieval function PlainSearch(D,Q,K) that computes similarity scores in the plaintext domain and returns the set of *K* document identifiers with the highest scores, denoted as ${\mathrm{Res}}_{K}^{*}=\left\{{\mathrm{fid}}_{\left(1\right)},\ldots ,{\mathrm{fid}}_{\left(K\right)}\right\}$, where the scores satisfy $s_{\left(1\right)}\geq s_{\left(2\right)}\geq \cdots \geq s_{\left(K\right)}\geq \ldots$ and $s_{\left(i\right)}$ is the score between ${\mathrm{fid}}_{\left(i\right)}$ and *Q*.

The scheme is said to be correct if and only if for all possible tuples (λ,D,Q,K) with 1≤K≤|D|, the following equality holds with probability 1:

$$\mathrm{Search}\left(I,T_{Q},K\right)={\mathrm{Res}}_{K}^{*}.$$

In other words, the set ${\mathrm{Res}}_{k}$ returned by the server’s encrypted search algorithm Search must be identical to the optimal plaintext result set ${\mathrm{Res}}_{K}^{*}$.

### 3.2. Leakage Functions

We adopt the honest-but-curious cloud server model, under which the cloud server faithfully follows the prescribed storage and retrieval protocols while attempting to infer additional information from all protocol-visible interactions, including the encrypted index, query trapdoors, search paths, and returned results. Active tampering, side-channel attacks, traffic analysis, and collusion attacks are beyond the scope of this work. Under this threat model, the security objective is to ensure that the cloud server learns no information beyond the explicitly defined leakage; in particular, it should be unable to recover the plaintext contents of the outsourced documents or queries, or derive any other secret information not permitted by the leakage profile. To formally characterize the information observable by the cloud server during protocol execution, we adopt the framework of explicit leakage functions [2,3]. This framework enables a principled trade-off between security and efficiency, allowing us to maintain the confidentiality of data content while explicitly delineating the information leakage tolerated by the scheme.

In searchable encryption, efficient retrieval typically necessitates revealing certain structural information and access patterns to the server. We formalize this leakage through a family of leakage functions

$$L=(L_{\mathrm{Setup}},L_{\mathrm{Search}})$$

that precisely describe the information leaked during different phases of the scheme.

- Setup Leakage $L_{\mathrm{Setup}}$: This captures the information revealed during system initialization and index construction. Typically, this includes global statistics such as the total number of documents and the dimensionality of their vector representations. If the scheme employs a tree-based index structure, this leakage may also encompass the topological information of the index tree.
- Search Leakage $L_{\mathrm{Search}}$: This models the information disclosed during the execution of a query. Its core components generally include:
  – Search Pattern: Reveals whether two queries are derived from the same plaintext keywords or query vector, i.e., their equivalence class.
  – Access Pattern: Reveals the set of document identifiers or specific index nodes accessed during query processing.
  – Result Pattern: Reveals the set of top-*K* document identifiers returned as the query result.
  For similarity ranking schemes, additional leakage, such as the Score Pattern, which reveals the relative ordering or comparison relations between query and document scores, will also occur.

In the section of security proof, we will instantiate a concrete leakage function family $L^{*}=\left(L_{\mathrm{Setup}}^{*},L_{\mathrm{Search}}^{*}\right)$ for our scheme and formally prove its security under $L^{*}$.

### 3.3. Security Definition

Let Σ denote the proposed scheme with an associated leakage function $L=(L_{\mathrm{Setup}},L_{\mathrm{Search}})$. We evaluate its security using the classic notion of Indistinguishability under Adaptive Chosen-Keyword Attack (IND2-CKA) [2,3].

The core intuition of this security model is as follows: Given two document collections, $D_{0}$ and $D_{1}$, that are indistinguishable with respect to the setup leakage $L_{\mathrm{Setup}}$, a computationally bounded adversary (e.g., an honest-but-curious cloud server) may adaptively issue a sequence of queries, each constrained to produce identical search leakage $L_{\mathrm{Search}}$ for both collections. Despite observing the encrypted index and query trapdoors, the adversary should be unable to distinguish whether the encrypted index corresponds to $D_{0}$ or $D_{1}$, gaining no information beyond what is explicitly permitted by L.

Formally, we define security by the following game.

**Definition 1**  (IND2-CKA Security)**.** *For any probabilistic polynomial-time (PPT) adversary A, we define the advantage experiment ${\mathrm{Exp}}_{\Sigma ,L}^{\mathrm{IND}2\text{-}\mathrm{CKA}}\left(A,\lambda \right)$ as follows:*

*1.*  *Setup: The challenger C runs $\mathrm{Setup}(1^{\lambda })$ to obtain the system secret key sk.*
*2.*  *Initialization: A outputs two plaintext document collections $D_{0}$ and $D_{1}$ subject to the constraint $L_{\mathrm{Setup}}\left(D_{0}\right)=L_{\mathrm{Setup}}\left(D_{1}\right)$.*
*3.*  *Phase 1: A may adaptively issue a polynomial number of queries. For the t-th query, A submits a query $Q^{\left(t\right)}$ such that $L_{\mathrm{Search}}\left(D_{0},Q^{\left(t\right)}\right)=L_{\mathrm{Search}}\left(D_{1},Q^{\left(t\right)}\right)$. C responds with the trapdoor $T_{Q^{\left(t\right)}}\leftarrow \mathrm{TrapdoorGen}\left(sk,Q^{\left(t\right)}\right)$.*
*4.*  *Challenge: C chooses a random bit $b\overset{\$}{\leftarrow }\left\{0,1\right\}$, computes the challenge index $I_{b}\leftarrow \mathrm{IndexBuild}\left(sk,D_{b}\right)$, and sends $I_{b}$ to A.*
*5.*  *Phase 2: A may continue to issue adaptive queries under the same constraint as in Phase 1.*
*6.*  *Guess: A outputs a guess bit $b^{\prime }$.*

*The experiment outputs 1 if $b^{\prime }=b$, and 0 otherwise. The advantage of A is defined as:*

$${\mathrm{Adv}}_{\Sigma ,L}^{\mathrm{IND}2\text{-}\mathrm{CKA}}\left(A,\lambda \right)=\left|\mathrm{Pr}\left[{\mathrm{Exp}}_{\Sigma ,L}^{\mathrm{IND}2\text{-}\mathrm{CKA}}\left(A,\lambda \right)=1\right]-\frac{1}{2}\right|.$$

*The scheme *Σ* is said to be **IND2-CKA secure** with respect to leakage L if for every PPT adversary A, the advantage ${\mathrm{Adv}}_{\Sigma ,L}^{\mathrm{IND}2\text{-}\mathrm{CKA}}\left(A,\lambda \right)$ is negligible in the security parameter λ.*

This definition ensures that even an adversary who adaptively chooses queries and observes their corresponding trapdoors and the encrypted index cannot distinguish the underlying plaintext collection with non-negligible advantage, thereby guaranteeing the semantic security of the scheme within the permitted leakage bounds.

## 4. Preliminaries

This section briefly introduces the fundamental techniques employed in our scheme. We first describe the Bag-of-Words model and dictionary construction for text representation. Then, we present the Latent Dirichlet Allocation model for extracting semantic topics, followed by the Word2Vec model for learning distributed word embeddings. Finally, we define the cosine similarity measure and the $\ell_{2}$ normalization process used for similarity computation.

### 4.1. Vocabulary, Bag-of-Words, and Tokenization

We adopt a standard Bag-of-Words (BoW) representation [35] to provide consistent inputs to the topic model. After applying an identical text normalization pipeline to the corpus (e.g., tokenization, lemmatization and stop-word removal), we build a global vocabulary (dictionary)

$$W=\left\{\left(w_{1},{\mathrm{ID}}_{1}\right),\ldots ,\left(w_{\left|W\right|},{\mathrm{ID}}_{\left|W\right|}\right)\right\},\;W\left[w_{i}\right]={\mathrm{ID}}_{i},$$

where |W| is the vocabulary size.

BoW representation. Each document *D* is encoded as a sparse term-frequency multiset over W:

BoW(D)={〈ID,tf〉∣ID∈[|W|]}.

This representation is used only as the input to LDA topic inference in our pipeline.

Token extraction. For a query text *Q*, we denote by wordsExtract(Q,W) the function that applies the same normalization as the corpus and outputs the in-vocabulary token IDs. In subsequent algorithms, the function is invoked through tokens←wordsExtract(Q,W), where $\mathrm{tokens}=[{\mathrm{ID}}_{1},{\mathrm{ID}}_{2},\ldots ,{\mathrm{ID}}_{m}]$ is a sequence of identifiers.

### 4.2. LDA Topic Model Outputs

We use Latent Dirichlet Allocation (LDA) [7] as a topic-level semantic representation. Given a corpus D with *n* documents, a vocabulary W, and a pre-defined topic number *t*, training/inference yields:

(Θ,Φ)←LDA.Model(t,D),

where the following outputs are used by our scheme.

- Document–topic matrix $\Theta \in R^{n\times t}$: The *i*-th row Θ[i] is the topic distribution vector of document $d_{i}$. We denote this vector by Θ(fid) when referring to a document identifier fid.
- Term–topic matrix $\Phi \in R^{|W|\times t}$: The *u*-th row Φ[u] captures the topic association of term $w_{u}$. We denote this vector by Φ(w) for a word *w*.
- Topic keyword lists KT.list: For each topic j∈[t], we rank terms by $\Phi_{:j}$ and keep the top-*m* entries:
  $$\mathrm{KT}.\mathrm{list}\left[j\right]=\{\left({\mathrm{ID}}_{1},s_{1}\right),\ldots ,\left({\mathrm{ID}}_{m},s_{m}\right)\},$$
  where *m* is tunable and $s_{i}$ is the corresponding topic score.

Θ(·) provides the semantic vectors for documents; Φ(·) provides topic association features for words; and KT.list helps topic-guided query expansion.

### 4.3. Word Embeddings for Similarity Filtering

We use Word2Vec [8] only as an embedding *lookup* for filtering expanded terms. Let

$$e:{\left\{0,1\right\}}^{*}\to R^{t}$$

be the embedding mapping that returns a *t*-dimensional vector for any in-vocabulary word *w*. In the sequel, e(w) denotes the embedding of *w*. Training details (CBOW/Skip-gram, negative sampling, etc.) are standard and are not required to understand the proposed scheme; they are therefore omitted here.

### 4.4. Cosine Similarity and $\ell_{2}$ Normalization

All semantic vectors used for similarity computation are $\ell_{2}$-normalized. For $x\in R^{t}$,

$$\hat{x}=\frac{x}{{∥x∥}_{2}},\;{∥x∥}_{2}=\sqrt{\sum_{i=1}^{t}x_{i}^{2}}.$$

Cosine similarity between u and v is

$$\mathrm{sim}\left(u,v\right)=\frac{u\cdot v}{{∥u∥}_{2}{∥v∥}_{2}}.$$

With $\ell_{2}$ normalization, cosine similarity reduces to an inner product:

$$\mathrm{sim}\left(\hat{u},\hat{v}\right)={\hat{u}}^{⊤}\hat{v}\in \left[-1,1\right].$$

Cosine similarity focuses on measuring the directional agreement between vectors and is invariant to scale changes, making it particularly suitable for similarity assessment and ranking in high-dimensional semantic spaces.

## 5. Plaintext Index Construction and Query Processing

This section presents the plaintext-domain pipeline underlying SES-HI. We describe: (i) a two-stage clustering routine that yields semantically compact leaf clusters of bounded size, (ii) an ω-ary balanced hierarchical index whose internal vectors serve as pruning upper bounds, (iii) a corpus-driven global pruning threshold τ, (iv) a topic-guided and embedding-filtered query vector construction, and (v) a pruned top-*K* traversal algorithm. These plaintext algorithms provide the foundation for the encrypted scheme presented in the next section.

**Figure 2:**
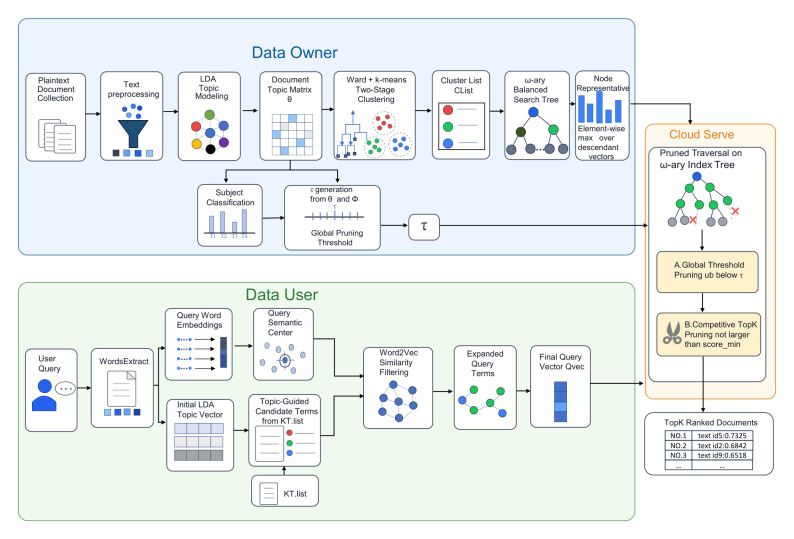
Overall workflow of plaintext index construction and query processing in SES-HI.

Figure 2 provides an overview of the plaintext index construction and query processing workflow of SES-HI. On the data-owner side, the document collection is preprocessed and transformed into LDA topic representations, after which Ward and *k*-means two-stage clustering is performed to construct an ω-ary balanced search tree and generate the global pruning threshold τ. On the data-user side, the original query is processed through topic-guided candidate selection and Word2Vec-based similarity filtering to obtain the final query vector. The cloud server then performs pruned traversal over the hierarchical index by combining global-threshold pruning and top-*K* competitive pruning, and finally returns the ranked documents.

### 5.1. Two-Stage Document Clustering via Ward and k-Means

Let each document *d* be represented by its topic vector $\Theta \left(d\right)\in R^{t}$. Our goal is to partition the corpus into a set of leaf clusters whose sizes are bounded by γ while maintaining semantic compactness in the topic space. We adopt a two-stage routine, WardKMeans, which uses Ward clustering to obtain robust candidate centers and then refines the partition using *k*-means initialized from these candidates. The parameter α>1 controls oversampling in the Ward stage to reduce sensitivity to poor initialization.

Given a document set *C*, WardKMeans recursively partitions *C* until each leaf cluster has size at most γ, producing $\mathrm{CList}=\{C_{1},\ldots ,C_{L}\}$ where $|C_{i}|\leq \gamma$. Each cluster $C_{i}$ is a list of tuples $C_{i}\left[j\right]=\left({\mathrm{fid}}_{i,j},v_{i,j}\right)$ with $v_{i,j}=\Theta \left[{\mathrm{fid}}_{i,j}\right]$. Algorithm 1 details the WardKMeans procedure.
**Algorithm 1** WardKMeans: Two-stage Document Clustering**Require:** Current document set *C*; document-topic matrix Θ; size threshold γ; target number of clusters *k*; Ward amplification factor α; global cluster list CList (initially empty)**Ensure:** Updated CList  1:**if** |C|≤γ **then**  2:    CList.add(C)  3:    **return**  4:**end if**  5:X←[Θ[fid]forfid∈C]  6:$k_{\mathrm{ward}}\leftarrow k\times \alpha$  7:$C_{\mathrm{ward}}\leftarrow \mathrm{Ward}\left(X,k_{\mathrm{ward}}\right)$  8:$C_{\mathrm{init}}\leftarrow k\text{-}\mathrm{means}\left(C_{\mathrm{ward}},k\right)$  9:$\left\{C_{1},\ldots ,C_{k}\right\}\leftarrow k\text{-}\mathrm{means}\left(X,C_{\mathrm{init}}\right)$10:**for** i=1 **to** *k* **do**11:    $\mathrm{WardKMeans}(C_{i},\Theta ,\gamma ,k,\alpha ,\mathrm{CList})$12:**end for**

The two-stage design is used to stabilize *k*-means under semantic-topic features: Ward provides a coarse but robust global structure (lines 5–7), and the compressed candidate centers $C_{\mathrm{init}}$ serve as a strong initialization for the refinement step (lines 8–9). This directly supports tighter and more stable node summaries in the subsequent hierarchical index.

**Figure 3:**
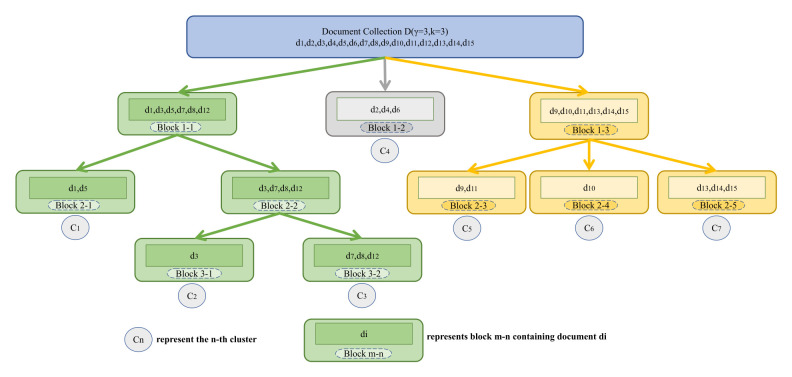
An example of two-stage clustering.

Figure 3 illustrates an example execution: with k=3,γ=3, an initial document set *C* containing 15 documents is recursively partitioned, resulting in 7 leaf clusters $\left\{C_{1},\ldots ,C_{7}\right\}$ that satisfy the size constraint.

### 5.2. Construction of ω-Ary Balanced Search Tree

We organize the cluster set $\mathrm{CList}=\{C_{1},\ldots ,C_{L}\}$ into an ω-ary balanced search tree, where each leaf node corresponds to one cluster. For any node *v* in this tree, we maintain a representative vector $u\left(v\right)\in R^{t}$, defined as the element-wise maximum over the topic vectors of all documents within the subtree rooted at *v*. This vector provides a semantic summary of its entire subtree. Crucially, it can be used to derive a provable upper bound for the relevance score between any document in the subtree and a given query, which forms the foundation of our pruning strategy.

Formally, the pruning mechanism relies on the following Upper-Bound Property. Assume that all document topic vectors are non-negative (as they represent topic distributions) and are $\ell_{2}$-normalized. For any non-negative query vector $Q_{\mathrm{vec}}⪰0$ and any document vector d residing in the subtree of node *v*, the inequality

$$d^{⊤}Q_{\mathrm{vec}}\leq u{\left(v\right)}^{⊤}Q_{\mathrm{vec}}$$

holds. The validity of this bound follows directly from the construction of u(v): since u(v) dominates d element-wise and $Q_{\mathrm{vec}}$ is component-wise non-negative, their inner product with u(v) necessarily bounds that with d.

**Figure 4:**
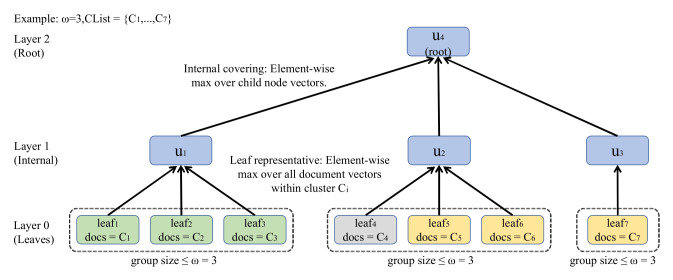
An Example of Index Tree Building.

Algorithm 2 constructs the tree bottom-up. The output is the root identifier root and a plaintext node table nodes_plain mapping each node identifier to (u,L), where *L* is either (i) a document list for a leaf node or (ii) a child-id list for an internal node. Figure 4 gives a balanced tree of height 3 as an example.
**Algorithm 2** Construction of ω-ary Balanced Search Tree**Require:** Document cluster list $\mathrm{CList}=\{C_{1},\ldots ,C_{L}\}$; branching factor ω; document-topic matrix Θ**Ensure:** Root node identifier root; plaintext node table nodes_plain  1:Initialize nodes_plain←∅, U←[]  2:**for** i=1 to *L* **do**                                                                                                         ▹ Construct leaf nodes  3:    Compute $u_{i}\leftarrow \max_{\mathrm{fid}\in C_{i}}\Theta \left[\mathrm{fid}\right]$  4:    ${\mathrm{leaf}}_{i}\leftarrow “\mathrm{leaf}”∥i$                                                                                  ▹ Generate unique leaf node identifier  5:    $\mathrm{nodes}\_\mathrm{plain}\left[{\mathrm{leaf}}_{i}\right]\leftarrow \left(u_{i},C_{i}\right)$                                                                          ▹ Store vector and document set  6:    $U.\mathrm{append}({\mathrm{leaf}}_{i})$  7:**end for**  8:h←1                                                                                                                         ▹ Counter for internal nodes  9:**while** |U|>1 **do**                                                                               ▹ Bottom-up construction of internal nodes10:    $U_{\mathrm{next}}\leftarrow \left[\;\right]$, m←|U|11:    **for** g=1 to ⌈m/ω⌉ **do**                                                                               ▹ Process groups of at most ω nodes12:        s←(g−1)×ω+1, e←min(g×ω,m)                                                         ▹ Start and end index of the group13:        G←{U[s],…,U[e]}                                                                                         ▹ Child nodes in the group14:        Compute $u_{\mathrm{parent}}\leftarrow \max_{\mathrm{child}\in G}\mathrm{nodes}\_\mathrm{plain}\left[\mathrm{child}\right].\mathrm{vector}$15:        ${\mathrm{inter}}_{h}\leftarrow “\mathrm{inter}”∥h$16:        $\mathrm{nodes}\_\mathrm{plain}\left[{\mathrm{inter}}_{h}\right]\leftarrow \left(u_{\mathrm{parent}},G\right)$17:        $U_{\mathrm{next}}.\mathrm{append}\left({\mathrm{inter}}_{h}\right)$, h←h+118:    **end for**19:    $U\leftarrow U_{\mathrm{next}}$20:**end while**21:root←U[0]22:**return** (root,nodes_plain)

### 5.3. Generation of Global Pruning Threshold τ

To enable coarse-grained pruning, we propose a corpus-driven global threshold τ. This threshold is derived from the empirical distribution of similarity scores between document topic vectors and a global topic-direction vector $v_{\mathrm{topic}}$. The direction vector $v_{\mathrm{topic}}$ is constructed by aggregating the representative strength of each topic from the topic matrix Φ. The specific value of τ is selected as a quantile of this similarity distribution, where the quantile level is dynamically mapped from the index structure ratio r=γ/ω. Intuitively, a larger *r* indicates a coarser tree structure (e.g., larger leaves or smaller fanout), warranting a more aggressive (higher) threshold for stronger pruning. Conversely, a smaller *r* calls for a more conservative threshold. This mapping p(r) serves as a lightweight mechanism to adaptively couple the pruning aggressiveness with the inherent granularity of the index, as formalized in Algorithm 3.
**Algorithm 3** Algorithm for Generating Global Pruning Threshold τ**Require:** Term-topic matrix $\Phi \in R^{|W|\times t}$; document-topic matrix $\Theta \in R^{n\times t}$; number of keywords *K*; quantile bounds $p_{\min},p_{\max}$; leaf cluster size γ; branching factor ω**Ensure:** Global pruning threshold τ  1:Initialize $m\leftarrow 0\in R^{t}$  2:**for** j=1 to *t* **do**  3:    ${\mathrm{vals}}_{j}\leftarrow [\Phi_{ij}\;\mathrm{for}\;i=1\;\mathrm{to}\;|W|]$  4:    $\mathrm{top}\_\mathrm{vals}\leftarrow \mathrm{TopK}({\mathrm{vals}}_{j},K)$  5:    $m_{j}\leftarrow \mathrm{Mean}\left(\mathrm{top}\_\mathrm{vals}\right)$  6:**end for**  7:$v_{\mathrm{topic}}\leftarrow m/{∥m∥}_{2}$  8:S←∅  9:**for** i=1 to *n* **do**10:    $s_{i}\leftarrow \Theta {\left[{\mathrm{fid}}_{i}\right]}^{⊤}v_{\mathrm{topic}}$11:    $S.\mathrm{insert}(s_{i})$12:**end for**13:$S_{\mathrm{sorted}}\leftarrow \mathrm{Sort}\left(S\right)$14:r←γ/ω15:$p\leftarrow p_{\min}+\left(p_{\max}-p_{\min}\right)\left(1-\exp\left(-r\right)\right)$16:$p^{\prime }\leftarrow \left\lceil \right|S_{\mathrm{sorted}}\left|\cdot p\right\rceil$17:$\tau \leftarrow S_{\mathrm{sorted}}\left[p^{\prime }\right]$18:**return** τ

Algorithm 3 operationalizes this idea in three concise stages: (i) computing and normalizing per-topic strengths to obtain $v_{\mathrm{topic}}$ (lines 1–7); (ii) calculating the similarity between every document and $v_{\mathrm{topic}}$ to build the empirical distribution (lines 8–12); and (iii) mapping the structure ratio *r* to a quantile position and extracting the corresponding similarity score as the final threshold τ (lines 13–17). By explicitly linking the threshold to the index geometry, this procedure ensures that the pruning strength is adaptively tuned to the specific configuration.

### 5.4. Query Vector Construction Algorithm

Short queries often yield sparse and unreliable topic evidence. To address this, we construct a semantically enriched query topic vector through a two-step expansion-and-reprojection procedure that synergistically leverages LDA and Word2Vec. This method enhances the query’s topic representation while carefully controlling the introduction of irrelevant semantic noise.

The core process, formalized in Algorithm 4, is as follows. First, an initial topic distribution $q_{\mathrm{topic}}$ and a Word2Vec-based semantic center $q_{w2v}$ are derived from the query. The top-ρ topics from $q_{\mathrm{topic}}$ are selected to guide the expansion. For each selected topic, relevant candidate keywords are retrieved from a pre-built topic-keyword list (KT.list). These candidates are then filtered by requiring their Word2Vec embeddings to have a cosine similarity not lower than a threshold δ to the query center $q_{w2v}$, ensuring semantic coherence. Finally, the original and filtered expansion terms are merged and reprojected onto the topic space using the term–topic matrix Φ to generate the final, enriched query vector $Q_{\mathrm{vec}}$.
**Algorithm 4** Query Expansion and Vector Construction using LDA and Word2Vec**Require:** Original query text $q_{\mathrm{text}}$; word embedding mapping e(·); keyword-topic list KT.list; term-topic matrix Φ; number of selected topics ρ; number of keywords per topic β; similarity threshold δ**Ensure:** Expanded query topic vector $Q_{\mathrm{vec}}$  1:$\mathrm{tokens}\leftarrow \mathrm{WordsExtract}(q_{\mathrm{text}})$  2:vecs←[e(w)forw∈tokensifw∈W]  3:$q_{w2v}\leftarrow \mathrm{Mean}\left(\mathrm{vecs}\right)$  4:$s\leftarrow 0\in R^{t}$  5:**for** each term w∈tokens **do**  6:    s←s+Φ(w)  7:**end for**  8:$q_{\mathrm{topic}}\leftarrow s/{∥s∥}_{2}$  9:$S_{\rho }\leftarrow \mathrm{TopIndices}\left(q_{\mathrm{topic}},\rho \right)$10:filtered_expanded←∅11:**for** each topic $t\in S_{\rho }$ **do**12:    $W_{t}\leftarrow \mathrm{KT}.\mathrm{list}\left[t\right]\left[:\;\beta \right]$13:    **for** each candidate term $w\in W_{t}$ **do**14:        **if** w∈W **then**15:           $s\leftarrow e{\left(w\right)}^{⊤}q_{w2v}$16:           **if** s≥δ **then**17:               filtered_expanded.add(w)18:           **end if**19:        **end if**20:    **end for**21:**end for**22:final_tokens←Unique(tokens∪filtered_expanded)23:$s\leftarrow 0\in R^{t}$24:**for** each term w∈final_tokens **do**25:    s←s+Φ(w)26:**end for**27:$Q_{\mathrm{vec}}\leftarrow s/{∥s∥}_{2}$28:**return** $Q_{\mathrm{vec}}$

This hybrid approach ensures the expanded query vector captures more comprehensive latent topic information while remaining semantically anchored to the user’s original intent, providing a robust foundation for accurate and efficient retrieval.

### 5.5. Index Tree Query Algorithm

Given the enriched query vector $Q_{\mathrm{vec}}$, the retrieval phase performs a pruned depth-first traversal of the ω-ary index tree to efficiently identify the top-*K* most relevant documents. The efficiency is achieved by leveraging two key pruning mechanisms that operate on the tree’s structure, where each internal node’s vector u(v) provides an element-wise maximum over its subtree, guaranteeing a valid semantic upper bound.

The traversal applies two pruning rules at each node *v* after computing its similarity upper bound $ub\left(v\right)=u{\left(v\right)}^{⊤}Q_{\mathrm{vec}}$:

(i) Global-threshold pruning: If ub(v)<τ, the entire subtree rooted at *v* is discarded, as no document within it can meet the minimum relevance threshold.
(ii) Competitive pruning: Once the candidate result set Res (maintained as a min-heap of capacity *K*) is full, if $ub\left(v\right)\leq {\mathrm{score}}_{\min}$ (the current *K*-th score), the subtree cannot improve the top-*K* results and is pruned.

When a leaf node is reached, the actual similarity scores between $Q_{\mathrm{vec}}$ and its constituent documents are computed. Documents with scores not lower than τ are used to update the candidate set Res.

**Figure 5:**
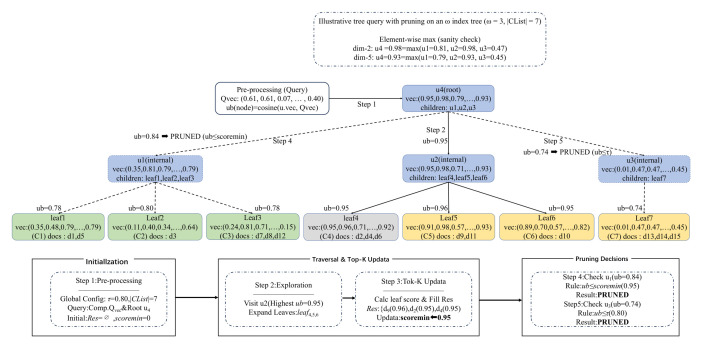
An example of index tree query algorithm.

This procedure is formalized in Algorithm 5 and illustrated with a concrete traversal example in Figure 5. By combining these two layers of pruning—one based on absolute relevance and the other on relative ranking—the search complexity is reduced from linear to sub-linear while preserving high retrieval accuracy, making the method scalable for large document collections.
**Algorithm 5** Pruned Query Algorithm on ω-ary Index Tree**Require:** Query vector $Q_{\mathrm{vec}}$; pruning threshold τ; number of returned documents *K*; node table nodes_plain; root node identifier root**Ensure:** Result set *R*, containing at most *K*(fid,score) pairs sorted in descending order of score  1:Res←∅                                                                                                                                 ▹ min-heap, capacity *K*  2:**if** root∈nodes_plain **then**  3:    DFS(root)  4:**end if**  5:R←SortDesc(Res); **return** *R*  6:**procedure** UpdateTopK((fid,s))  7:    **if** |Res|<K **then**  8:        Res.insert((fid,s))  9:    **else if** s>minScore(Res) **then**10:        Res.removeMin(); Res.insert((fid,s))11:    **end if**12:**end procedure**13:**procedure** DFS(*v*)14:    **if** v∉nodes_plain **then return**15:    **end if**16:    node←nodes_plain[v]17:    u←node.vector; $ub\leftarrow u^{⊤}Q_{\mathrm{vec}}$18:    θ←τ                                                                                                                        ▹ effective pruning threshold19:    **if** |Res|=K **then**20:        θ←max(τ,minScore(Res))21:    **end if**22:    **if** ub<θ **then return**23:    **end if**24:    **if** node is an internal node **then**25:        **for** each child c∈node.children **do**26:           DFS(*c*)27:        **end for**28:    **else**29:        **for** each $(\mathrm{fid},d_{\mathrm{vec}})\in \mathrm{node}.\mathrm{docs}$ **do**30:           $s\leftarrow d_{\mathrm{vec}}^{⊤}Q_{\mathrm{vec}}$31:           **if** s≥τ **then**32:               UpdateTopK((fid,s))33:           **end if**34:        **end for**35:    **end if**36:**end procedure**

## 6. Concrete Construction of SES-HI

Building upon the plaintext index construction and query processing algorithms presented in Section 5, this section presents the complete SES-HI scheme. We employ an encryption scheme Π that supports secure inner-product computation (e.g., enhanced asymmetric scaler product encrytion (EASPE) [36,37], or inner product encryption (IPE) [38]) as the underlying cryptographic black box. The proposed construction is modular with respect to the underlying inner-product encryption primitive, and its concrete efficiency, leakage profile, and scalability depend on the selected instantiation. The scheme Π typically consists of four algorithms: Π.Setup generates the key, Π.Enc encrypts a vector, Π.TokenGen generates a query token, and Π.Compute computes the inner product of two vectors. Utilizing these primitives and based on the system model defined in Section 3.1, we construct the following four algorithms, which together constitute our SES-HI scheme.

- $\mathrm{Setup}(1^{\lambda })$: On input of the security parameter λ, it invokes $\Pi .\mathrm{Setup}(1^{\lambda })$ to generate the vector encryption key $sk_{\Pi }$. It also generates a symmetric key $k_{\mathrm{doc}}$ for document content encryption. The algorithm outputs the system secret key $SK=(sk_{\Pi },k_{\mathrm{doc}})$.
- IndexBuild(SK,D): On input of the secret key SK and the document collection D, it first invokes the plaintext tree construction algorithm (Algorithm 2) to obtain the root node identifier root and the plaintext node table nodes_plain. Subsequently, it traverses each node in nodes_plain and encrypts its vector fields using Π: for the node representative vector u, it computes the ciphertext $\tilde{u}\leftarrow \Pi .\mathrm{Enc}\left(sk_{\Pi },u\right)$; if the node is a leaf, it also encrypts each document vector $d_{\mathrm{vec}}$ within it to obtain ${\tilde{d}}_{\mathrm{vec}}$. The encrypted node structure is stored in the ciphertext node table nodes_cipher, preserving the original tree topology. Finally, the algorithm outputs the encrypted index I=(root,nodes_cipher). The detailed pseudocode is provided in Algorithm 6. The Global Pruning Threshold τ is generated independently using the method described in Section 5.3. It is considered a public parameter shared with the data user for the Search algorithm. In addition, to ensure full confidentiality, the actual content of each document can be encrypted using the symmetric key $k_{\mathrm{doc}}$ (e.g., via AES). The resulting ciphertexts can be stored alongside the encrypted document vectors in the leaf nodes of nodes_cipher without affecting the index structure or the search logic.

**Algorithm 6** IndexBuild: Encrypted Index Construction
**Require:** Secret key $SK=(sk_{\Pi },k_{\mathrm{doc}})$; plaintext node table nodes_plain (from Algorithm 2)**Ensure:** Root node identifier root; ciphertext node table nodes_cipher
  1:Initialize nodes_cipher←∅  2:**for** each node identifier v∈nodes_plain **do**  3:    u←nodes_plain[v].vector, $\tilde{u}\leftarrow \Pi .\mathrm{Enc}\left(sk_{\Pi },u\right)$, $\mathrm{nodes}\_\mathrm{cipher}\left[v\right].\mathrm{vector}\leftarrow \tilde{u}$  4:    **if** *v* is an internal node **then**  5:        nodes_cipher[v].children←nodes_plain[v].children  6:    **else**  7:        Initialize docs_enc←[]  8:        **for** each $\left(\mathrm{fid},d_{\mathrm{vec}}\right)\in \mathrm{nodes}\_\mathrm{plain}\left[v\right].\mathrm{docs}$ **do**  9:           ${\tilde{d}}_{\mathrm{vec}}\leftarrow \Pi .\mathrm{Enc}\left(sk_{\Pi },d_{\mathrm{vec}}\right)$, $\mathrm{docs}\_\mathrm{enc}.\mathrm{append}(\left(\mathrm{fid},{\tilde{d}}_{\mathrm{vec}}\right))$10:        **end for**11:        nodes_cipher[v].docs←docs_enc12:    **end if**13:
**end for**
14:**return** (root,nodes_cipher)

- $\mathrm{TrapdoorGen}(SK,q_{\mathrm{text}})$: On input of the secret key SK and a query text $q_{\mathrm{text}}$, it first invokes the query expansion algorithm (Algorithm 4) to transform $q_{\mathrm{text}}$ into an enhanced query vector $Q_{\mathrm{vec}}$. Then, it uses $\Pi .\mathrm{TokenGen}(sk_{\Pi },Q_{\mathrm{vec}})$ to generate the query trapdoor $T_{Q}$. The algorithm outputs $T_{Q}$.
- $\mathrm{Search}(I,T_{Q},K)$: On input of the encrypted index I, the query trapdoor $T_{Q}$, the global pruning threshold τ, and the number of documents to return *K*, this algorithm follows the same control flow and pruning logic as the plaintext tree query algorithm (Algorithm 5), with the only modification that all similarity computations $u^{⊤}Q_{\mathrm{vec}}$ are replaced by $\Pi .\mathrm{Compute}(u,T_{Q})$. Specifically:
  – For any node *v*, its upper bound score is computed as $ub\leftarrow \Pi .\mathrm{Compute}(\tilde{u},T_{Q})$, where $\tilde{u}$ is the ciphertext of the node’s representative vector.
  – The pruning conditions remain unchanged: if ub<τ or (the candidate set is full and $ub\leq {\mathrm{score}}_{\min}$), then the branch is pruned.
  – For each document ciphertext ${\tilde{d}}_{\mathrm{vec}}$ in a leaf node, the score is computed as $\mathrm{score}\leftarrow \Pi .\mathrm{Compute}({\tilde{d}}_{\mathrm{vec}},T_{Q})$, and the candidate result set is updated according to the same rules as in the plaintext algorithm.

The algorithm finally outputs the top-*K* document identifiers along with their scores, sorted in descending order.

Since the output of Π.Compute is consistent with the plaintext inner product computation, the correctness of the scheme is guaranteed, i.e., the encrypted search results are identical to the plaintext search results.

## 7. Leakage Functions and Security Proof

This section formalizes the information leakage of our proposed encryption scheme and proves its indistinguishability under adaptive chosen-keyword attacks (IND2-CKA). We first present the concrete leakage functions based on the scheme’s structure and algorithm flow, and then complete the security proof based on the security of the underlying encryption scheme Π, which supports secure inner-product computation.

### 7.1. Leakage Instantiation

Based on the index structure and algorithm flow described in Section 6, we instantiate a concrete leakage function family:

$$L^{\mathrm{Tree}}=\left(L_{\mathrm{Setup}}^{\mathrm{Tree}},L_{\mathrm{Search}}^{\mathrm{Tree}}\right).$$

#### 7.1.1. Setup Leakage $L_{\mathrm{Setup}}^{\mathrm{Tree}}$

Given a plaintext database D, the server can observe the overall structural information of the index during the initialization phase. This information is determined by our index construction algorithm (Algorithm 6) and mainly includes: the total number of documents |D|; the dimension *t* of document vectors (i.e., the number of topics); index tree parameters: the tree height *h* and the branching factor ω; the number of leaf nodes (document clusters) *L* and the upper bound γ on the number of documents per leaf node.

Thus, the setup leakage function is formally defined as:

$$L_{\mathrm{Setup}}^{\mathrm{Tree}}\left(D\right):=\left(|D|,t,h,\omega ,L,\gamma \right).$$

#### 7.1.2. Search Leakage $L_{\mathrm{Search}}^{\mathrm{Tree}}$

For a query *Q*, while executing the Search algorithm for tree traversal and pruning, the server can observe the following information:

- Search Pattern (SP): Reveals whether two queries correspond to the same query vector. Since identical query vectors produce exactly the same tree traversal path, the server can identify the equivalence class of queries.
- Access Pattern (AP): Reveals which nodes (including internal and leaf nodes) are visited during the tree traversal and which branches are pruned. This reflects the access path of the query in the index tree.
- Result Pattern (RP): Reveals the set of top-*K* document identifiers $R_{Q}$ returned by the query. This information must be returned to the user by the server.
- Score Pattern (ScoP): For each node *u* visited during the traversal, the server obtains the inner-product similarity score between that node’s representative vector (or document vector) and the query vector using the secure computation functionality of the underlying scheme Π. These scores are directly used to decide pruning and final result ranking.

To formalize, let the set of all node and document vectors in the index tree be ${\left\{x_{i}\right\}}_{i\in I}$. For a query *Q* (corresponding to query vector q), the score pattern is defined as the sequence of all computed inner products ($x_{i}^{⊤}q$). Therefore, the search leakage function is defined as:

$$L_{\mathrm{Search}}^{\mathrm{Tree}}\left(D,Q\right):=\left({\mathrm{SP}}_{Q},{\mathrm{AP}}_{Q},{\mathrm{RP}}_{Q},{\mathrm{ScoP}}_{Q}\right).$$

In summary, the complete leakage function of the scheme is:

$$L^{\mathrm{Tree}}:=\left(L_{\mathrm{Setup}}^{\mathrm{Tree}},L_{\mathrm{Search}}^{\mathrm{Tree}}\right).$$

### 7.2. Security Proof

Based on the above leakage functions, we present the security theorem for our scheme.

**Theorem 1.**  *Let the underlying encryption scheme Π be a semantically secure scheme that supports inner-product computation. Then the SES-HI scheme satisfies IND2-CKA security (Definition 1) with respect to the leakage function $L^{\mathrm{Tree}}$.*

**Proof.**  We prove the theorem by a series of hybrid games that reduce the advantage of any PPT adversary A attacking our scheme to the advantage of breaking the underlying scheme Π.

Step 1:  Slotification of the index.

For the encrypted index constructed by the proposed scheme, each node (including the root, internal nodes, and leaf nodes) and each document in a leaf node is represented by a vector, and all these vectors are encrypted using Π.Enc. Let the total number of encrypted vectors in the index be *M*. We can view these encrypted items as *M* independent “slots”, denoted as ${\mathrm{slot}}_{1},{\mathrm{slot}}_{2},\ldots ,{\mathrm{slot}}_{M}$. Each slot ${\mathrm{slot}}_{i}$ stores a ciphertext $C_{i}$ of a vector $x_{i}$.

In the IND2-CKA experiment, the challenger receives two databases $D_{0}$ and $D_{1}$ such that $L_{\mathrm{Setup}}^{\mathrm{Tree}}\left(D_{0}\right)=L_{\mathrm{Setup}}^{\mathrm{Tree}}\left(D_{1}\right)$. Therefore, for b∈{0,1}, the plaintext index built from $D_{b}$ has exactly the same tree structure (same h,ω,n,γ), and only the specific vector values in the slots may differ. Let $x_{i}^{\left(b\right)}$ denote the plaintext vector corresponding to slot ${\mathrm{slot}}_{i}$ when the challenge bit is *b*.

Step 2:  Define the hybrid game sequence ${\left\{{\mathrm{Game}}_{j}\right\}}_{j=0}^{M}$.

We define M+1 hybrid games:

- ${\mathrm{Game}}_{0}$: All *M* slots contain ciphertexts of $x_{i}^{\left(0\right)}$. This game simulates the case where the challenge bit b=0.
- ${\mathrm{Game}}_{M}$: All *M* slots contain ciphertexts of $x_{i}^{\left(1\right)}$. This game simulates the case where b=1.
- ${\mathrm{Game}}_{j}$ (1≤j≤M−1): The first *j* slots (${\mathrm{slot}}_{1},\ldots ,{\mathrm{slot}}_{j}$) contain ciphertexts from $D_{1}$ (i.e., encryptions of $x_{i}^{\left(1\right)}$), while the remaining M−j slots contain ciphertexts from $D_{0}$ (i.e., encryptions of $x_{i}^{\left(0\right)}$).

Clearly, the advantage of adversary A in distinguishing ${\mathrm{Game}}_{0}$ from ${\mathrm{Game}}_{M}$ is exactly its advantage in the IND2-CKA experiment.

Step 3:  Indistinguishability of adjacent hybrids.  

We claim that for any j∈{1,…,M}, no PPT adversary can distinguish ${\mathrm{Game}}_{j-1}$ from ${\mathrm{Game}}_{j}$ with non-negligible advantage. If such an adversary A exists, we can construct an adversary B that breaks the semantic security of the underlying scheme Π.

Adversary B interacts with the challenger of Π in a security experiment, aiming to distinguish the encryptions of two plaintexts $x_{j}^{\left(0\right)}$ and $x_{j}^{\left(1\right)}$. B simulates the IND2-CKA experiment environment for A as follows:

1. Simulate system parameters: B obtains the public parameters from Π’s challenger and forwards them to A.
2. Simulate the challenge index: When the challenge index needs to be generated, B produces ciphertexts for each slot *i*:
  - For i<j: B asks Π’s challenger for an encryption of $x_{i}^{\left(1\right)}$.
  - For i>j: B asks Π’s challenger for an encryption of $x_{i}^{\left(0\right)}$.
  - For i=j: B submits the challenge plaintext pair $\left(x_{j}^{\left(0\right)},x_{j}^{\left(1\right)}\right)$ to Π’s challenger and receives the challenge ciphertext $C_{j}^{*}$, which is used as the ciphertext for ${\mathrm{slot}}_{j}$.
  If Π’s challenger encrypts $x_{j}^{\left(0\right)}$, then B simulates ${\mathrm{Game}}_{j-1}$; if it encrypts $x_{j}^{\left(1\right)}$, then B simulates ${\mathrm{Game}}_{j}$.
3. Simulate queries: When A adaptively submits a query *Q*, the experiment requires that $L_{\mathrm{Search}}^{\mathrm{Tree}}\left(D_{0},Q\right)=L_{\mathrm{Search}}^{\mathrm{Tree}}\left(D_{1},Q\right)$. This implies that for all slots *i*, $\langle x_{i}^{\left(0\right)},q\rangle =\langle x_{i}^{\left(1\right)},q\rangle$, where q is the query vector corresponding to *Q*. Therefore, B can request from Π’s challenger a token for vector q and use it to execute Π.Compute on all slot ciphertexts (including $C_{j}^{*}$), obtaining identical inner-product results. Using these results, B can perfectly simulate the pruning logic, score pattern, access pattern, and result pattern of the Search algorithm and return the simulated view to A.
4. Output: Finally, B outputs A’s guess bit for the challenge index. If A can distinguish ${\mathrm{Game}}_{j-1}$ from ${\mathrm{Game}}_{j}$, then B can distinguish the encryptions of $x_{j}^{\left(0\right)}$ and $x_{j}^{\left(1\right)}$ under Π with the same advantage.

By the semantic security assumption of Π, the advantage of B is negligible, and hence the advantage ${\mathrm{Adv}}_{j}$ of A in distinguishing adjacent hybrids is also negligible.

Step 4:  Summation of advantages.

The overall advantage of adversary A in the original IND2-CKA experiment satisfies:

$${\mathrm{Adv}}_{\Sigma ,L^{\mathrm{Tree}}}^{\mathrm{IND}2-\mathrm{CKA}}\left(A,\lambda \right)=\left|\mathrm{Pr}\left[{\mathrm{Game}}_{0}\left(A\right)=1\right]-\mathrm{Pr}\left[{\mathrm{Game}}_{M}\left(A\right)=1\right]\right|\leq \sum_{j=1}^{M}{\mathrm{Adv}}_{j}.$$

Since *M* is a polynomial function of the database size and each ${\mathrm{Adv}}_{j}$ is negligible, the sum remains a negligible function of the security parameter λ.

In conclusion, for any PPT adversary A, the advantage ${\mathrm{Adv}}_{\Sigma ,L^{\mathrm{Tree}}}^{\mathrm{IND}2-\mathrm{CKA}}\left(A,\lambda \right)$ is negligible, which completes the proof of the theorem.    □

## 8. Comprehensive Evaluation and Discussion

This section presents a comprehensive examination of the proposed scheme, including theoretical complexity analysis, experimental evaluation, Ablation Study Analysis, and discussion. Specifically, first, a formal study of the computational complexity establishes the foundational efficiency characteristics of our scheme. Subsequently, extensive experiments compare the scheme against state-of-the-art baselines, quantifying its advantages in retrieval accuracy and speed. Finally, we discuss the results, elucidate the scheme’s advantages and limitations, and outline promising directions for future research.

### 8.1. Theoretical Complexity Analysis

This section systematically analyzes the theoretical time complexity of the proposed scheme and four representative baseline schemes, including EPSMR [9], CASE-SSE [10], PBINS [30], and SSS KG [31], across three phases: index construction, trapdoor generation, and query execution. EPSMR and CASE-SSE employ EASPE-style secure inner-product computation [36,37] as the underlying encrypted inner-product primitive, while PBINS relies on semantic vectors, K-means-based private bins, range-encoded bitmaps, and encrypted bitmap access. SSS KG represents documents as a knowledge graph and uses an adjacency-list-style encrypted structure combined with MRSE-based semantic matching. The following parameters are used: *n* denotes the number of documents, *t* denotes the semantic vector dimension used by the proposed scheme, EPSMR, PBINS, and SSS KG, *m* denotes the vocabulary dimension used by CASE-SSE, γ denotes the maximum number of documents per leaf node, ω denotes the tree branching factor, ρ denotes the number of topics selected for query expansion, β denotes the number of candidate keywords per topic, *k* denotes the number of clusters or classes generated by K-means in clustering-based schemes, such as CASE-SSE and PBINS, and *K* denotes the number of returned results. For PBINS, *k* denotes the number of private bins generated by K-means, and each private bin contains approximately n/k documents on average. For SSS KG, G=(V,E,L) denotes the document knowledge graph, where *L* represents vertex and edge labels or semantic information, $G_{Q}=\left(V_{Q},E_{Q},L_{Q}\right)$ denotes the query graph, and |DB[v]| denotes the number of encrypted records or relationships associated with a matched query vertex *v*. Common output materialization costs, such as returning the final *K* identifiers, are omitted unless explicitly stated.

#### 8.1.1. Index Construction Phase

The proposed scheme first partitions the *n* documents into clusters of size at most γ, which serve as leaf nodes. An index tree is then built bottom-up by aggregating these leaf nodes with a branching factor ω. The number of leaf nodes is approximately n/γ, and the number of internal nodes is approximately n/(γω). Thus, the total number of tree nodes is approximately n(ω+1)/(γω). Each node and each document are represented by a *t*-dimensional vector and encrypted using EASPE, whose single-vector encryption cost is $O(t^{2})$. Therefore, the overall index construction complexity of the proposed scheme is:

$$T_{\mathrm{index}}^{\mathrm{Ours}}=O\left(\left[\frac{n(\omega +1)}{\gamma \omega }+n\right]t^{2}\right).$$

EPSMR adopts a similar tree-based encrypted index structure. Since it also encrypts the document vectors and tree-node vectors with the same EASPE-style primitive, its index construction complexity is:

$$T_{\mathrm{index}}^{\mathrm{EPSMR}}=O\left(\left[\frac{n(\omega +1)}{\gamma \omega }+n\right]t^{2}\right).$$

CASE-SSE maps documents to *m*-dimensional term-frequency vectors, constructs a category-oriented tree or clustered index, and encrypts the corresponding index vectors using EASPE. If *k* denotes the number of classes or clusters, its index construction complexity is:

$$T_{\mathrm{index}}^{\mathrm{CASE}}=O\left(km^{2}\right).$$

PBINS first represents the *n* documents as *t*-dimensional semantic vectors, and then uses K-means to partition these documents into *k* private bins. Each private bin is associated with a centroid vector, and the average number of documents in each private bin is approximately n/k. In PBINS, both the document vectors and the private-bin centroid vectors are regarded as part of the searchable encrypted index and need to be encrypted or key-encapsulated. Since encrypting a single *t*-dimensional vector incurs $O(t^{2})$ time cost, the index construction complexity of PBINS can be expressed as:

$$T_{\mathrm{index}}^{\mathrm{PBINS}}=O\left(\left(n+k\right)t^{2}\right).$$

Here, *n* corresponds to the encryption cost of document vectors, while *k* corresponds to the encryption cost of private-bin centroid vectors. The construction of the range-encoded bitmap index is performed over the documents inside each private bin. Under the fixed two-digit base-10 range encoding setting, the number of bitmap columns in each bin is constant, and thus this part only introduces a linear organizational cost with respect to the number of document entries. Therefore, it is omitted from the dominant encrypted-index construction term.

SSS KG first extracts or constructs a knowledge graph G=(V,E,L) from the *n* documents, where *L* represents vertex and edge labels or semantic information. After the graph is obtained, its vertices and edges are organized into computable semantic representations. Since the subsequent encrypted index is built over the graph structure rather than directly over the original document list, the construction cost depends mainly on the scale of the extracted graph. For each vertex, SSS KG stores encrypted metadata and adjacency information. For each edge, it generates an encrypted semantic representation for relation matching. If each graph-related vector is represented in a *t*-dimensional space and the encryption or tokenized matrix-vector operation over one vector costs $O(t^{2})$, the index construction complexity of SSS KG can be written as:

$$T_{\mathrm{index}}^{\mathrm{SSS}\;\mathrm{KG}}=O\left(\left(|V\right|+\left|E|\right)t^{2}\right).$$

This expression indicates that the construction cost of SSS KG is determined by the number of vertices and edges extracted from the document collection. When the documents contain many entities and relations, both |V| and |E| increase, leading to a higher encrypted-index construction cost.

#### 8.1.2. Trapdoor Generation Phase

The proposed scheme first performs query expansion. It selects ρ topics from the keyword-topic list, takes the top β candidate keywords per topic, and filters them using Word2Vec. This step has complexity O(ρβt). Then, the expanded *t*-dimensional query vector is encrypted using EASPE with complexity $O(t^{2})$. The total trapdoor generation complexity is:

$$T_{\mathrm{token}}^{\mathrm{Ours}}=O\left(\rho \beta t+t^{2}\right).$$

EPSMR does not perform the controlled query expansion used in the proposed scheme. It directly encrypts the original query vector with EASPE. Therefore, its trapdoor generation complexity is:

$$T_{\mathrm{token}}^{\mathrm{EPSMR}}=O\left(t^{2}\right).$$

CASE-SSE expands the query by computing semantic similarity between the query term and candidate words in the vocabulary, selecting the top β nearest terms, and then encrypting the *m*-dimensional query vector. The expansion cost is O(βm), and the EASPE encryption cost is $O(m^{2})$. Thus:

$$T_{\mathrm{token}}^{\mathrm{CASE}}=O\left(\beta m+m^{2}\right).$$

PBINS first obtains the *t*-dimensional query vector corresponding to the query text, and then encrypts or key-encapsulates this query vector to generate the trapdoor. Since encrypting a single *t*-dimensional query vector incurs $O(t^{2})$ time cost, the trapdoor generation complexity of PBINS is:

$$T_{\mathrm{token}}^{\mathrm{PBINS}}=O\left(t^{2}\right).$$

In this analysis, the cost of generating the plaintext query embedding is not included, because the comparison focuses on the encrypted trapdoor construction after the query vector has been obtained.

SSS KG first transforms the query request into a query graph $G_{Q}=\left(V_{Q},E_{Q},L_{Q}\right)$, where $L_{Q}$ represents query-side vertex and edge labels or semantic information. Without separately counting the natural-language query-graph extraction cost, the trapdoor generation phase mainly processes the vertices and edges in the query graph. For each query vertex and query edge, the scheme generates the corresponding encrypted token information. Since the encryption or MRSE-style trapdoor generation over a *t*-dimensional vector costs $O(t^{2})$, the trapdoor generation complexity of SSS KG can be expressed as:

$$T_{\mathrm{token}}^{\mathrm{SSS}\;\mathrm{KG}}=O\left(\left(\right|V_{Q}\left|+\right|E_{Q}\left|\right)t^{2}\right).$$

This complexity shows that the trapdoor generation cost of SSS KG depends on the size of the query graph. A simple query graph leads to a relatively small trapdoor cost, whereas a complex query graph with more entities and relations introduces higher token generation overhead.

#### 8.1.3. Query Execution Phase

The proposed scheme starts from the root node and performs a depth-first traversal on the ω-ary encrypted index tree. The tree height is $h=\lceil \log_{\omega }\left(n/\gamma \right)\rceil$. Each visited node requires one encrypted inner-product computation with cost O(t). Thus, the tree traversal part has complexity $O(t\log_{\omega }\left(n/\gamma \right))$. After reaching a leaf node, the scores of at most γ documents within that leaf are computed, each requiring O(t). Therefore, the leaf processing cost is O(tγ), and the overall query execution complexity is:

$$T_{\mathrm{search}}^{\mathrm{Ours}}=O\left(t\log_{\omega }\left(n/\gamma \right)+t\gamma \right).$$

EPSMR employs the same tree-based retrieval structure and pruning strategy. Therefore, its query execution complexity is:

$$T_{\mathrm{search}}^{\mathrm{EPSMR}}=O\left(t\log_{\omega }\left(n/\gamma \right)+t\gamma \right).$$

CASE-SSE searches the target class node in an AVL-tree-style structure. The tree search depth is O(logk), and each node visit requires an *m*-dimensional encrypted inner-product computation. Therefore, the search complexity is:

$$T_{\mathrm{search}}^{\mathrm{CASE}}=O\left(m\log k\right).$$

After locating the target class, the top-*K* results are returned from the associated inverted list. Since this operation is independent of the encrypted tree traversal, it is usually omitted from the main asymptotic search cost.

In the query execution phase, PBINS first uses the trapdoor to determine the closest private bin. Since the number of private-bin centroids is *k*, and each comparison between the query vector and a centroid requires an O(t) similarity computation, the nearest-bin selection cost is O(kt). After the closest private bin is determined, PBINS accesses the range-encoded bitmap columns selected by the trapdoor and evaluates a fixed Boolean expression over the recovered bitmap columns. At the protocol level, the number of accessed bitmap columns is constant. However, in an actual implementation, each accessed bitmap column has a length proportional to the number of documents in the selected private bin. Since each private bin contains approximately n/k documents on average, bitmap recovery, Boolean evaluation, and candidate collection introduce an implementation-level cost that is proportional to the accessed bitmap length. Considering the *t*-dimensional score-related operations in the practical implementation, this part can be written as O((n/k)t). Therefore, the practical query execution complexity of PBINS can be expressed as:

$$T_{\mathrm{search}}^{\mathrm{PBINS}}=O\left(\left(k+\frac{n}{k}\right)t\right).$$

This explains why PBINS has constant protocol-level bitmap access but still exhibits non-negligible practical query time due to nearest-bin selection, bitmap recovery, Boolean operations, and candidate collection.

SSS KG performs encrypted matching over the adjacency-list-style encrypted knowledge graph. The server does not scan all documents, but only traverses the encrypted adjacency records associated with the matched query vertex. Let |DB[v]| denote the number of encrypted records or relationships associated with the matched vertex *v*. For each accessed adjacency record, the server performs node matching, relation matching, and a *t*-dimensional semantic comparison. Therefore, the query execution complexity for a simple query graph can be written as:

$$T_{\mathrm{search}}^{\mathrm{SSS}\;\mathrm{KG}}=O\left(|DB\left[v\right]|t\right).$$

For complex query graphs, the search process typically involves multiple query vertices and query edges. In this case, the total cost depends on the cumulative number of adjacency-list entries visited during graph matching.

Table 1 summarizes the theoretical time complexities of all compared schemes. In the index construction phase, the proposed scheme and EPSMR have similar complexity because both construct a multi-layer encrypted tree and encrypt both document vectors and internal node vectors. CASE-SSE has a relatively compact construction form with respect to the number of class nodes, but its dependence on the high-dimensional vocabulary space leads to an $m^{2}$ encryption cost. For PBINS, the index construction cost is mainly caused by the encryption or key-encapsulation of both document vectors and private-bin centroid vectors. Since the number of document vectors is *n* and the number of centroid vectors generated by K-means is *k*, its dominant encrypted-index construction cost is $O(\left(n+k\right)t^{2})$. Although the range-encoded bitmap construction is also required, the number of bitmap columns in each bin is fixed under the two-digit base-10 encoding setting, and thus this part only contributes a linear organizational cost. For SSS KG, the index construction cost is determined by the size of the knowledge graph extracted from the document collection. A larger number of vertices and edges leads to a higher cost of graph-vector encryption and adjacency-list-style index construction, which is summarized as $O((|V|+\left|E|\right)t^{2})$.

In the trapdoor generation phase, EPSMR has the lowest theoretical complexity because it directly encrypts the query vector without additional semantic expansion. The proposed scheme and CASE-SSE introduce query expansion, which increases trapdoor generation cost but improves the semantic expressiveness of the query. PBINS only encrypts or key-encapsulates the obtained *t*-dimensional query vector, resulting in $O(t^{2})$ complexity. The nearest-bin selection is treated as part of the query execution phase rather than trapdoor generation. SSS KG generates encrypted tokens for the query graph, and its trapdoor generation cost depends on the number of vertices and edges in the query graph, namely $O((|V_{Q}\left|+\right|E_{Q}\left|\right)t^{2})$.

In the query execution phase, the proposed scheme and EPSMR follow a logarithmic tree traversal plus bounded leaf-level scoring process. CASE-SSE has a compact tree-search complexity, but its direct truncation from the class-related inverted list may reduce retrieval accuracy. PBINS first compares the query vector with *k* private-bin centroids and then accesses the selected range-encoded bitmap columns inside the selected bin. Since the selected bin contains approximately n/k documents on average, the practical query cost is O((k+n/k)t), where the first term corresponds to nearest-bin selection and the second term corresponds to bitmap recovery, Boolean evaluation, and candidate collection inside the selected bin. SSS KG achieves sublinear search by visiting only the adjacency-list entries associated with the matched query vertex. Its query cost is O(|DB[v]|t). Therefore, the measured wall-clock time of PBINS and SSS KG should be interpreted together with the number of private bins, the average private-bin size, the knowledge-graph scale, the adjacency-list length, and the unified timing protocol used in the experiments.

### 8.2. Experimental Evaluation

This section presents a systematic experimental evaluation of the proposed scheme. The experiments are conducted on the real-world TREC dataset [39] using Python 3.10, with the hardware platform being an Intel^®^ Core™ i5-14600KF processor and 32 GB of RAM. To comprehensively assess performance, four representative and recent semantic-aware searchable encryption schemes, including EPSMR, CASE-SSE, PBINS, and SSS KG, are selected as baselines. A quantitative comparison is performed across four key dimensions: index construction time, trapdoor generation time, query execution time, and retrieval accuracy.

#### 8.2.1. Experimental Setup

We sampled 50,000 documents along with their standard query topics from the TREC dataset to form the test corpus. All schemes are executed in the same software and hardware environment to ensure comparability. The five schemes under comparison are summarized as follows:

- EPSMR: A tree-indexed searchable encryption scheme based on document clusters. It employs *k*-means clustering and the LDA semantic model, without query expansion.
- CASE-SSE: A cluster-based searchable encryption scheme. It uses Word2Vec for query expansion, and its index structure is an AVL tree.
- SES-HI: Employs two-stage clustering (Ward + *k*-means) to construct an ω-ary balanced search tree. It integrates LDA and Word2Vec for semantic vector generation and selective query expansion.
- PBINS: A private-bin-based encrypted semantic search scheme. It partitions document semantic vectors into *k* private bins using K-means, and supports efficient retrieval through range-encoded bitmap indexes and encrypted bitmap access.
- SSS KG: A knowledge-graph-based encrypted semantic search scheme. It extracts a knowledge graph from outsourced documents and organizes encrypted graph records through an adjacency-list-style structure, enabling semantic matching based on encrypted node and relation information.

For parameters shared by multiple schemes, such as the semantic vector dimension *t*, we use the same values to ensure comparability. Scheme-specific structural parameters, such as the maximum number of documents per leaf node γ, the branching factor ω, the number of K-means clusters or private bins *k*, and the graph-related quantities in SSS KG, are configured according to the corresponding index structure and are reported in the relevant experiments.

It is important to note that the original CASE-SSE scheme operates in a high-dimensional term-frequency space (dimension *m*), where *m* is typically much larger than the semantic vector dimension *t* used in topic models (i.e., m≫t). Directly encrypting in the full *m*-dimensional space would incur prohibitively high memory and time costs. To ensure a fair and feasible comparison, we adopt a semantic embedding substitution strategy in our implementation: each document is represented as a low-dimensional semantic vector $z_{d}\in R^{t}$ (where t≪m). For all subsequent complexity analysis and performance comparisons, the semantic dimension *t* is uniformly used for equivalent evaluation. This approach controls resource consumption while maintaining query accuracy and retrieval efficiency.

#### 8.2.2. Index Building Time Comparison

The index building time is primarily influenced by the number of documents *n* and the semantic vector dimension *t*. In this subsection, we compare the construction cost of the five schemes under varying document scales and vector dimensions. The parameter sensitivity of γ and ω is discussed later in the ablation study.

**Figure 6:**
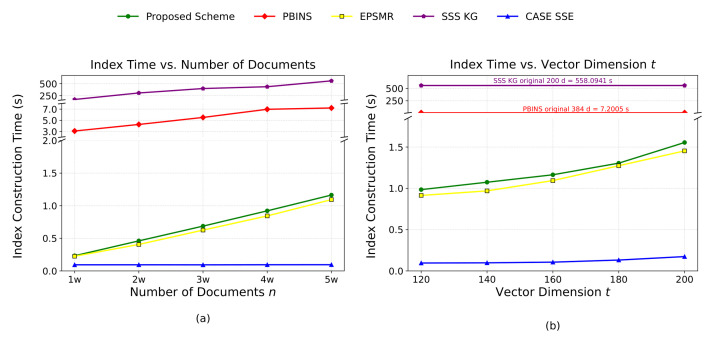
Comparison of the index building time. (**a**) Index construction time for different document quantities. (**b**) Index construction time for different vector dimensions.

Document Scale *n* (Figure 6a). As *n* increases from 10,000 to 50,000, the construction time of both the proposed scheme and EPSMR grows approximately linearly, consistent with theoretical analysis. The construction time of CASE-SSE remains largely unaffected by *n*, as its index structure depends only on the predefined number of clusters *k*. The proposed scheme takes slightly longer than EPSMR, due to its finer-grained two-stage clustering strategy, which generates more document clusters (leaf nodes), thereby increasing the total number of nodes in the index tree and the associated encryption overhead. PBINS has a clearly higher index construction time than the proposed scheme and EPSMR, but remains lower than SSS KG. This is because PBINS relies on SBERT-based Transformer sentence embeddings and private-bin partitioning, and both semantic representation and bin organization introduce additional construction cost. SSS KG has the highest index construction time and increases significantly with *n*, because it needs to construct and encrypt knowledge graph structures, including entities, relations, adjacency information, and MRSE-related index components. Therefore, its construction overhead is much larger than that of ordinary vector-tree index schemes. The axis-break design in Figure 6a is used because the construction time of SSS KG is in a different order of magnitude from the other schemes.

Vector Dimension *t* (Figure 6b). For the tunable lightweight schemes, namely the proposed scheme, EPSMR, and CASE-SSE, index construction time increases as *t* grows. This is consistent with the increasing encryption and vector-processing cost caused by higher-dimensional semantic representations. The proposed scheme and EPSMR have similar growth trends, while the proposed scheme is slightly higher due to the larger number of tree nodes produced by the two-stage clustering process. CASE-SSE remains the lowest because its index structure is simpler and its node scale is limited. PBINS and SSS KG are plotted as fixed reference baselines rather than dimension-varying curves. PBINS is shown at its original 384-dimensional SBERT setting, while SSS KG is shown at its original 200-dimensional KG embedding/MRSE setting. Their fixed reference points indicate that PBINS introduces a moderate additional construction cost, whereas SSS KG has a substantially higher construction overhead due to KG construction and encrypted graph-index generation.

In summary, the results are consistent with the theoretical complexity analysis. The construction time of the proposed scheme and EPSMR increases with document scale and vector dimension because both schemes rely on cluster-based tree indices and encrypted vector representations. PBINS incurs higher construction cost due to Transformer-based semantic embedding and private-bin organization. SSS KG has the largest construction overhead because of KG construction and encrypted graph-index generation. Although CASE-SSE has the lowest construction time, its retrieval precision and ranking quality are significantly weaker, as shown in the retrieval accuracy experiments. Therefore, the proposed scheme achieves a more balanced trade-off between index construction cost and retrieval quality.

#### 8.2.3. Trapdoor Generation Time Comparison

This experiment uses the standard query topics from the TREC dataset as input. Since all schemes transform query text into semantic vectors or structured semantic representations for processing, trapdoor generation time is mainly influenced by the semantic vector dimension *t* and by the query-side semantic processing mechanism. Furthermore, as SES-HI and CASE-SSE incorporate query expansion, the impact of the number of expansion terms β must also be examined.

**Figure 7:**
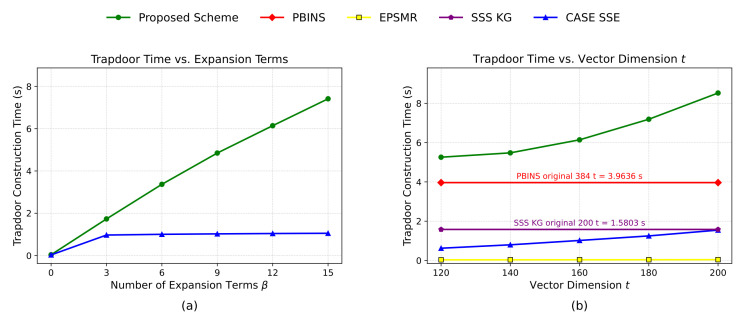
Comparison of the trapdoor generation time. (**a**) The influence of different word expansion quantities on the time required for constructing the trapdoor. (**b**) The impact of different vector dimensions on the construction of the trapdoor.

Number of Expansion Terms β (Figure 7a). Figure 7a focuses on the schemes whose trapdoor generation process is directly affected by the number of expansion terms. The trapdoor generation time of the proposed scheme increases almost linearly as β grows. This is because the proposed query expansion procedure performs topic-guided candidate selection and Word2Vec-based semantic filtering. As more expansion terms are considered, more similarity computations between candidate terms and the query center vector are required, and the additional cost becomes proportional to the expansion scale. CASE-SSE also uses semantic expansion, but its growth is much gentler because its expansion process mainly relies on selecting similar terms from the pre-trained Word2Vec vocabulary. Therefore, the proposed scheme introduces higher trapdoor construction overhead than CASE-SSE in exchange for more strictly controlled semantic expansion and better suppression of low-relevance expansion noise.

Vector Dimension *t* (Figure 7b). As *t* increases, the trapdoor generation time of the tunable vector-based schemes increases monotonically. This is consistent with the theoretical conclusion that the encryption operation over a *t*-dimensional vector introduces an $O(t^{2})$ cost. Specifically, the proposed scheme has the highest cost among the tunable lightweight schemes because it performs semantic filtering before encrypting the expanded query vector. CASE-SSE has a moderate cost, while EPSMR has the lowest trapdoor generation time because it directly encrypts the original query vector without query expansion. In addition, PBINS and SSS KG are shown as fixed reference baselines under their original configurations rather than dimension-varying curves. The horizontal lines are used only for visual comparison with the tunable lightweight schemes; they do not indicate that PBINS and SSS KG were evaluated at all tested dimensions from 120 to 200. PBINS is reported at its original 384-dimensional semantic representation, with a trapdoor generation time of 3.9636 s. SSS KG is reported at its original 200-dimensional knowledge-graph or MRSE-related setting, with a trapdoor generation time of 1.5803 s.These fixed baselines indicate that the trapdoor cost of PBINS and SSS KG should be interpreted together with their original semantic representation and structural matching mechanisms, rather than as part of the same low-dimensional sensitivity curve.

The experimental results for the trapdoor generation phase are consistent with the theoretical complexity analysis in Section 8.1. The number of expansion terms β mainly affects schemes employing explicit query expansion, where semantic filtering introduces an approximately linear overhead. The semantic vector dimension *t* imposes an $O(t^{2})$ cost on the encryption operations of vector-based schemes. Consequently, the proposed scheme has higher trapdoor generation cost because it combines topic-guided expansion, semantic filtering, and vector encryption. However, this additional cost is introduced on the user side and is used to obtain a more reliable semantic query representation. PBINS and SSS KG provide useful fixed reference points, but their trapdoor generation times are affected by their original Transformer-style semantic representation or knowledge-graph-based query processing settings, and therefore should not be directly interpreted as tunable low-dimensional curves.

#### 8.2.4. Query Execution Time Comparison

According to the theoretical analysis in Section 8.1, the query execution time is mainly affected by the document scale *n* and the semantic vector dimension *t*. This subsection compares the query efficiency of the five schemes under varying document scales and vector dimensions. The sensitivity of the structural parameters γ and ω is further analyzed in the ablation study.

**Figure 8:**
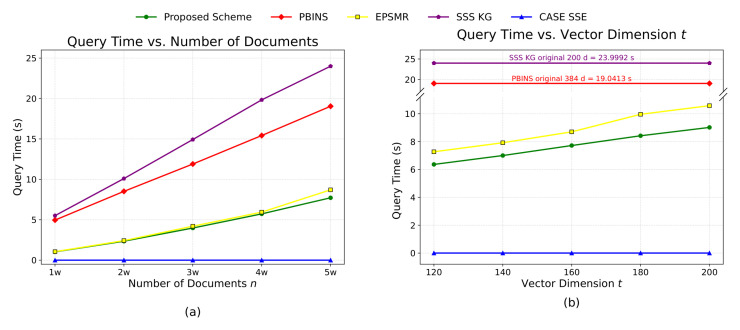
Comparison of the query execution time. (**a**) Query time for different numbers of documents. (**b**) Query time for vectors of different dimensions.

Document Scale *n* (Figure 8a). As *n* increases from 10,000 to 50,000, the query time of the proposed scheme and EPSMR increases steadily. The proposed scheme is consistently faster than EPSMR, indicating that the two-stage clustering strategy provides better semantic coherence and enables more effective pruning during tree traversal. CASE-SSE remains almost unchanged with respect to *n*, because its query process mainly depends on a preset cluster structure rather than the full document scale. However, this low time cost should be interpreted together with its much weaker retrieval accuracy reported previously.

PBINS and SSS KG also show increasing query time as *n* grows. PBINS is slower than the proposed scheme and EPSMR, mainly because its private-bin organization and Transformer-based semantic representation introduce additional matching and candidate processing overhead. SSS KG has the highest query time among the five schemes. This is reasonable because its query process relies on structured knowledge graph matching, including node matching, relation matching, and encrypted graph-index traversal. Therefore, although SSS KG provides a richer structural semantic representation, its query execution cost is substantially higher in this experimental setting.

Vector Dimension *t* (Figure 8b). For the tunable schemes, namely the proposed scheme, EPSMR, and CASE-SSE, query time increases as the semantic vector dimension *t* grows. This is consistent with the theoretical analysis that similarity computation and encrypted vector operations introduce higher cost under larger dimensions. The proposed scheme remains faster than EPSMR across all tested dimensions, showing that its pruning mechanism can reduce unnecessary node evaluation even when the vector dimension increases. CASE-SSE has the lowest query time because of its simpler cluster-level search process, but its retrieval effectiveness is also much lower.

PBINS and SSS KG are not plotted as full dimension-varying curves in this experiment. Instead, they are shown as fixed reference baselines under their original configurations. PBINS is shown at its original 384-dimensional Transformer sentence embedding setting, while SSS KG is shown at its original 200-dimensional knowledge graph embedding or MRSE setting. The results show that both fixed baselines have higher query time than the proposed scheme and EPSMR under the tested lightweight dimensions. In particular, SSS KG incurs the largest query cost, which is mainly caused by graph-based semantic matching and encrypted relation traversal.

In conclusion, the experimental results are consistent with the theoretical analysis. The query time of the proposed scheme and EPSMR increases with document scale and vector dimension, while the proposed scheme remains faster due to more effective pruning enabled by its two-stage clustering structure. PBINS introduces additional query overhead because of its Transformer-based representation and private-bin search process. SSS KG has the highest query time because of knowledge graph matching and encrypted graph traversal. Although CASE-SSE has the lowest query time, its retrieval accuracy is significantly weaker. Therefore, the proposed scheme achieves a more balanced trade-off between query efficiency and retrieval effectiveness.

#### 8.2.5. Retrieval Accuracy Comparison

To systematically evaluate the retrieval quality of each scheme, this experiment compares the accuracy of the five schemes under identical evaluation conditions, considering four dimensions: document scale *n*, semantic vector dimension *t*, number of expansion terms β, and number of returned documents *K*.

**Figure 9:**
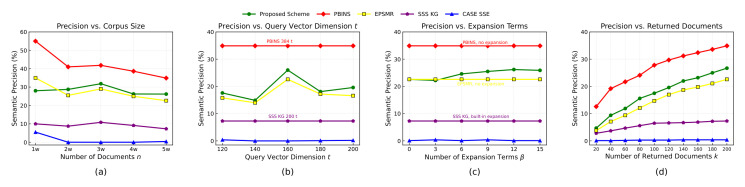
Comparison of the retrieval accuracy. (**a**) Accuracy rates corresponding to different numbers of documents. (**b**) Accuracy rates corresponding to different vector dimensions. (**c**) Accuracy rates corresponding to different numbers of expanded words. (**d**) Accuracy rates corresponding to different numbers of returned documents.

In addition to the overall retrieval-accuracy trends shown in Figure 9, we further report the top-ranked retrieval quality using Precision@10, Precision@20, and NDCG@10. The curves in Figure 9 mainly reflect the general Precision@200 tendency under different experimental conditions, whereas the additional metrics in Table 2 and Table 3 provide a finer evaluation of the returned results at shallower ranks. Precision@K measures the proportion of relevant documents among the top-*K* returned results, while NDCG@10 further considers the ranking positions of relevant documents within the top-10 list.

The results in Table 2 show that PBINS generally obtains strong top-ranked retrieval quality, especially at 1w and 5w, which is consistent with the advantage of Transformer-based sentence embeddings. Nevertheless, the proposed scheme remains competitive among lightweight non-Transformer encrypted semantic retrieval schemes. For example, under 5w, the proposed scheme achieves 5.40% Precision@10 and 4.90% Precision@20, both higher than EPSMR, SSS KG, and CASE-SSE. The NDCG@10 results further show that the proposed scheme can maintain stable ranking quality, while SSS KG and CASE-SSE are consistently weaker under this ordinary text retrieval setting. Table 3 further confirms that the proposed scheme is less sensitive to low-dimensional semantic representation than EPSMR and CASE-SSE. In particular, at 180 dimensions, the proposed scheme achieves the best results among the tunable lightweight schemes in terms of Precision@10 and NDCG@10. PBINS is reported only at its original 384-dimensional setting, and SSS KG is reported only at its original 200-dimensional setting; therefore, they should be interpreted as fixed reference baselines rather than complete dimension-sensitivity curves.

Document Scale *n* (Figure 9a). As *n* increases, the retrieval precision of all five schemes exhibits different trends. PBINS maintains the highest precision over different document scales, mainly because it relies on high-capacity SBERT-based Transformer sentence embeddings, such as paraphrase MiniLM, which can capture stronger contextual semantic information than lightweight topic-vector representations. The proposed scheme is lower than PBINS, but it remains consistently higher than EPSMR, SSS KG, and CASE-SSE. This shows that the two-stage clustering strategy and semantic-filtering-based query expansion in the proposed scheme are still effective under lightweight semantic models. The higher accuracy over EPSMR is primarily attributed to the improved semantic consistency within leaf nodes and the query expansion process that suppresses low-relevance expansion noise. SSS KG obtains relatively low precision in this plain-text retrieval setting because its knowledge graph query matching is sensitive to entity extraction, relation extraction, and query-graph matching quality; therefore, the structural advantage of knowledge graph representation cannot always be fully exploited on ordinary text retrieval data. CASE-SSE remains close to zero, indicating that its context-aware semantic extension and index structure are difficult to use for stably capturing semantically relevant documents under this dataset and query setting.

Vector Dimension *t* (Figure 9b). The curves of the proposed scheme and EPSMR show a non-monotonic trend as the semantic vector dimension *t* varies, indicating that lightweight semantic spaces are sensitive to the choice of dimensionality. A moderate dimension can improve semantic representation, whereas an excessively small dimension tends to discard useful topic information and an excessively large dimension tends to introduce noise. The proposed scheme outperforms EPSMR under most dimensions, showing that its clustering-based hierarchical index and semantic-filtering-based query expansion improve the effectiveness of low-dimensional semantic representations. PBINS and SSS KG are not dimension-varying curves in this experiment, but are plotted according to their original fixed configurations as reference baselines. PBINS uses the default 384-dimensional sentence vector of SBERT paraphrase MiniLM, and is therefore shown as a PBINS original 384 d baseline. SSS KG uses the 200-dimensional knowledge graph embedding or MRSE matrix setting reported in its original design, and is therefore shown as an SSS KG original 200 d baseline. Although PBINS achieves a higher fixed-baseline precision due to its stronger Transformer representation, this result should not be interpreted as a fair dimension-sensitivity comparison under the same low-dimensional encrypted vector condition.

Number of Expansion Terms β (Figure 9c). As β increases, the precision of the proposed scheme generally increases and reaches a favorable level around a moderate expansion scale. This confirms that proper query expansion can enlarge semantic coverage and improve the chance of matching relevant documents. Compared with EPSMR, the proposed scheme benefits from a semantic filtering mechanism that removes low-relevance expansion terms and therefore achieves a better balance between expansion gain and noise control. EPSMR and PBINS do not adopt the same controllable expansion-word mechanism as the proposed scheme, and thus they are shown as no-expansion baselines in this figure. SSS KG has its own KG-based semantic extension during query processing, but this extension is not implemented by generating a tunable number of expansion terms. Instead, it expands the matching space of the structured query graph through node type matching and MRSE-protected edge similarity matching. CASE-SSE also involves context-aware semantic extension, but its precision remains low across the tested expansion range, suggesting that its expansion and indexing mechanism is less stable for this retrieval setting.

Number of Returned Documents *K* (Figure 9d). As *K* increases, the precision of most schemes increases because a larger returned set is more likely to contain relevant documents. PBINS still maintains the highest growth curve, indicating that Transformer semantic embeddings provide stronger semantic ranking ability in top-*K* retrieval. The proposed scheme achieves a higher precision and a stronger growth trend than EPSMR, SSS KG, and CASE-SSE, which demonstrates that its query expansion strategy and hierarchical pruning-aware index continue to improve the hit rate when the return scale expands. EPSMR also benefits from a larger return set, but its accuracy remains lower because its semantic representation and pruning structure are less discriminative. SSS KG grows slowly, implying that the recall ability of knowledge graph structure matching is limited under this experimental setting. CASE-SSE only increases slightly and remains the lowest overall.

Overall, Figure 9 provides the macroscopic trend of retrieval precision under different document scales, dimensions, expansion-term numbers, and returned-document numbers, while Table 2 and Table 3 provide a more detailed view of the top-ranked retrieval quality. The combined results indicate that PBINS benefits from stronger Transformer embeddings, whereas the proposed scheme achieves more stable and competitive performance under lightweight encrypted semantic retrieval settings. The advantages of the proposed scheme are especially evident when compared with EPSMR, SSS KG, and CASE-SSE in the top-ranked Precision@10, Precision@20, and NDCG@10 results.

In summary, PBINS obtains the highest retrieval precision because it adopts high-capacity SBERT-based Transformer sentence representations. However, the goal of the proposed scheme is not to exceed the accuracy upper bound provided by strong Transformer embeddings, but to achieve stable and competitive precision under lightweight encrypted semantic retrieval conditions. Compared with EPSMR, SSS KG, and CASE-SSE, the proposed scheme consistently provides better precision by combining two-stage clustering, semantic-filtering-based query expansion, and a pruning-aware hierarchical index structure. These mechanisms enhance semantic consistency, reduce expansion noise, and improve the discriminative capability of encrypted retrieval, thereby achieving a more practical trade-off between efficiency and precision.

### 8.3. Ablation Study Analysis

#### 8.3.1. Parameter Sensitivity Analysis

In addition to the main comparison under document scale and vector dimension, we further evaluate the sensitivity of the proposed index structure to two important structural parameters, namely the maximum number of documents per leaf γ and the branching factor ω. This analysis helps clarify how the hierarchical index behaves under different tree configurations and whether the additional structure introduced by the proposed scheme leads to unstable construction overhead. Since CASE-SSE, PBINS, and SSS KG do not adopt the same γ and ω control mechanism as the proposed scheme, this parameter sensitivity experiment mainly compares the proposed scheme with EPSMR.

**Figure 10:**
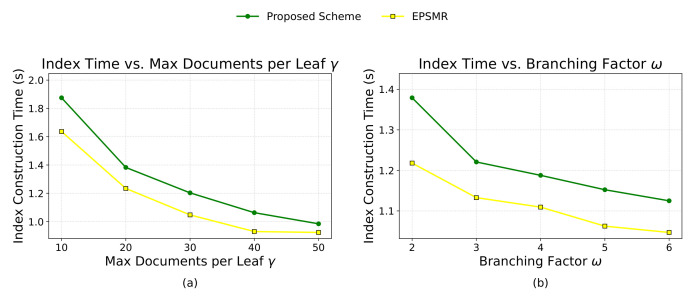
Parameter sensitivity analysis of index building time with respect to γ and ω. (**a**) The impact of the maximum number of documents corresponding to each leaf node on the indexing construction time. (**b**) The impact of the number of different branches in the tree on the time required for index construction.

Leaf Size γ (Figure 10a). As γ increases, each leaf node can contain more documents, so the number of generated leaf nodes decreases. Therefore, the total number of encrypted tree nodes and the corresponding encryption operations are reduced. As shown in Figure 10a, the index construction time of both the proposed scheme and EPSMR decreases monotonically with increasing γ. EPSMR is slightly faster because its *k*-means-based clustering structure produces fewer or more compact nodes. The proposed scheme is slightly slower because the two-stage clustering strategy produces a more fine-grained hierarchical structure, but this additional cost supports better semantic consistency and retrieval precision.

Branching Factor ω (Figure 10b). As ω increases, each internal node can have more children, which reduces the tree height and the number of internal nodes. Therefore, the construction time decreases as ω increases. As shown in Figure 10b, both curves decrease and then gradually become stable, indicating that further increasing ω brings diminishing returns. EPSMR remains slightly faster, while the proposed scheme maintains a comparable construction cost. This demonstrates that the proposed index structure is not overly sensitive to ω and can maintain stable construction efficiency under different branching-factor settings.

In addition to the index-building cost, we further evaluate how γ and ω affect query execution time. Since PBINS, SSS KG, and CASE-SSE do not adopt the same γ and ω control mechanism as the proposed hierarchical index, this part mainly compares the proposed scheme with EPSMR.

**Figure 11:**
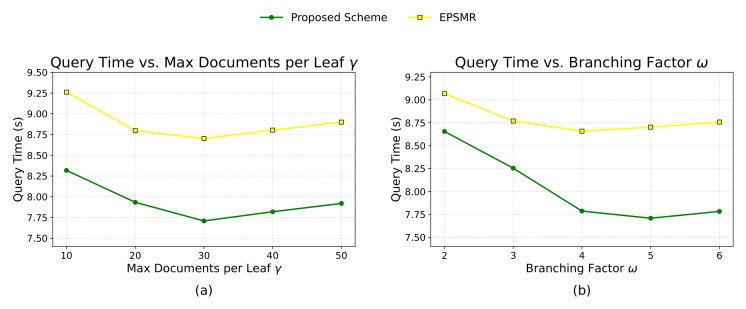
Parameter sensitivity analysis of query execution time with respect to γ and ω. (**a**) The impact of the maximum number of documents corresponding to each leaf node on the query time. (**b**) The influence of the number of different branches in the tree on the query time.

Leaf Size γ and Query Time (Figure 11a). As γ increases, the query time of both the proposed scheme and EPSMR first decreases and then slightly increases. When γ is small, the index contains more leaf nodes and the tree traversal cost is relatively high. Increasing γ makes the tree flatter and reduces traversal overhead, so the query time decreases. However, when γ becomes too large, each leaf node contains more candidate documents, increasing intra-leaf similarity computation and causing a slight rebound in query time. The proposed scheme remains faster than EPSMR across all tested γ values, which indicates that the two-stage clustering strategy provides more compact semantic groups and supports more effective pruning.

Branching Factor ω and Query Time (Figure 11b). As ω increases, the query time of both schemes generally decreases at first and then becomes stable or slightly rebounds. A larger ω reduces the tree height and the number of traversal levels, thereby lowering query time. However, when ω is too large, each internal node has more child branches, which tends to weaken the discriminative effect of hierarchical pruning and increase the number of candidate branches evaluated at each level. Therefore, the query time does not continue to decrease indefinitely. The proposed scheme is consistently faster than EPSMR, further confirming that its hierarchical index provides stronger pruning ability under different branching-factor settings.

#### 8.3.2. Ablation Study on the Pruning Threshold τ

The pruning threshold τ is introduced to control branch access during hierarchical tree search. In this experiment, τ is selected according to different quantiles of the similarity distribution between the query vector and node representations. A lower τ corresponds to a more relaxed pruning condition, allowing more nodes to enter subsequent traversal and reducing the risk of discarding potentially relevant branches. In contrast, a higher τ imposes stricter pruning, which can reduce the number of visited nodes and accelerate query execution, but may also remove relevant candidate branches too early. Therefore, τ directly controls the trade-off between retrieval accuracy and query efficiency.

**Figure 12:**
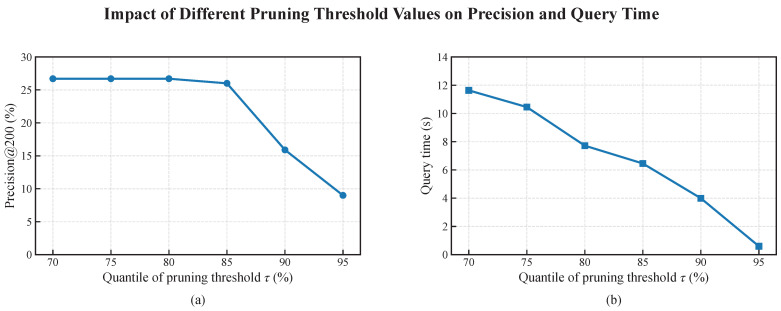
Impact of different pruning threshold values on Precision@200 and query time. (**a**) The impact of different pruning thresholds on accuracy. (**b**) The impact of different pruning thresholds on query time.

Effect on Precision@200 (Figure 12a). As the quantile of τ increases from 70% to 80%, Precision@200 remains stable at 26.70%, indicating that moderate pruning does not noticeably harm retrieval accuracy. When τ increases to 85%, Precision@200 slightly decreases to 26.00%, suggesting that the pruning condition becomes stricter but still preserves most relevant branches. However, when τ is further increased to 90% and 95%, Precision@200 drops sharply to 15.90% and 9.00%, respectively. This shows that an excessively high pruning threshold removes too many candidate branches before they can be evaluated, resulting in insufficient candidate coverage and a significant loss of retrieval precision.

Effect on Query Time (Figure 12b). The query time decreases continuously as the quantile of τ increases. Specifically, the query time is reduced from 11.6342 s at the 70% quantile to 7.7188 s at the 80% quantile, and further decreases to 0.5986 s at the 95% quantile. This trend is consistent with the role of τ: a higher pruning threshold filters out more branches, thereby reducing the number of visited nodes and similarity computations during tree search. However, the fastest setting is not necessarily the best choice, because the sharp reduction in query time at very high τ values is accompanied by a substantial loss of retrieval precision.

Overall, the 80% quantile provides the best balance between retrieval precision and query efficiency in this experiment. At this setting, Precision@200 remains at 26.70%, the same as the 70% and 75% settings, while the query time is reduced to 7.7188 s. Although higher thresholds such as 90% and 95% further accelerate query execution, they cause a sharp degradation in Precision@200. Therefore, we select the 80% quantile as the default setting of τ in the subsequent experiments, since it achieves effective pruning without sacrificing retrieval accuracy.

#### 8.3.3. Ablation Study on the Expansion Similarity Threshold δ

The expansion similarity threshold δ is used to control the semantic reliability of query expansion terms. In this ablation study, *T* denotes the number of topic-level expansion terms, and *W* denotes the number of word-level or Word2Vec-based expansion terms. The threshold δ denotes the expansion similarity filtering threshold and determines whether a candidate expansion term is sufficiently similar to the query semantic center and can therefore be retained. A smaller δ tends to introduce more expansion terms and irrelevant semantic noise, whereas a larger δ filters out weakly related candidates but also increases the risk of discarding useful complementary semantics.Therefore, this ablation study evaluates how different combinations of *T*, *W*, and δ affect Precision@200.

The first row in Table 4 corresponds to the no-expansion setting, where T=0, W=0, and δ=0. In this case, Precision@200 is 22.60%. This result indicates that the original query vector can already capture part of the query semantics, but the semantic coverage is still limited because the query usually contains only a small number of terms. Therefore, properly selected expansion terms can further improve retrieval effectiveness.

When δ=0, the expansion terms are added without effective semantic filtering. Under T=3 and W=3,4,5, Precision@200 decreases to 20.50%, 20.70%, and 20.60%, respectively, all of which are lower than the no-expansion baseline. This demonstrates that query expansion without similarity-based filtering tends to introduce weakly related or noisy terms, causing semantic drift in the expanded query representation. Therefore, merely increasing the number of expansion terms is not sufficient; the semantic quality of the selected terms must also be controlled.

After introducing a positive similarity threshold, the retrieval precision improves significantly. For example, when T=3 and W=5, increasing δ from 0 to 0.5 improves Precision@200 from 20.60% to 25.50%. The best result is achieved when T=4, W=4, and δ=0.5, where Precision@200 reaches 26.70%. This confirms that similarity-based filtering can effectively suppress noisy expansion terms while preserving useful semantic complements, thereby improving the discriminative ability of the expanded query.

However, an excessively strict threshold or an excessive number of expansion terms does not further improve performance. Under T=4 and W=4, Precision@200 increases from 23.90% at δ=0.35 to 26.70% at δ=0.5, but decreases to 25.80% when δ is further increased to 0.55. This suggests that too high a threshold may discard some useful expansion terms. Similarly, when both *T* and *W* are increased to 5, Precision@200 reaches 25.80% at δ=0.5, but still remains lower than the best setting of T=4, W=4, and δ=0.5. These results show that both the number of expansion terms and the similarity threshold should be jointly controlled to avoid semantic noise and over-filtering.

Overall, the ablation results show that the optimal setting in this experiment is T=4, W=4, and δ=0.5, which achieves the highest Precision@200 of 26.70%. Compared with the no-expansion baseline, this setting improves Precision@200 by 4.10 percentage points. Therefore, we adopt T=4, W=4, and δ=0.5 as the default query expansion configuration in the subsequent experiments. This setting provides a favorable balance between semantic coverage and noise suppression.

#### 8.3.4. Sensitivity Analysis of LDA Prior Parameters

LDA contains two important prior parameters that affect topic representation. To avoid conflict with the existing notation in this paper, we denote the document-topic prior as $\lambda_{DT}$, which corresponds to the gensim LDA hyperparameter named alpha. This parameter controls the sparsity of the topic distribution for each document. A smaller $\lambda_{DT}$ means that a document is more concentrated on a few topics, whereas a larger $\lambda_{DT}$ allows a document to cover more topics. We denote the topic-word prior as $\lambda_{TW}$, which corresponds to the gensim LDA hyperparameter named eta. This parameter controls the sparsity of the word distribution within each topic. A smaller $\lambda_{TW}$ means that each topic is characterized by fewer high-probability words, whereas a larger $\lambda_{TW}$ makes each topic cover more words.

In general, LDA can use the auto setting to learn prior parameters automatically. Therefore, this paper adopts $\lambda_{DT}=\mathrm{auto}$ and $\lambda_{TW}=\mathrm{auto}$ as the baseline configuration. To examine the influence of topic-model prior parameters on the quality of topic-guided query expansion in Algorithm 4, we further test several common small prior values around $10^{-3}$ to $10^{-2}$, with an additional larger setting of 0.05.

The results in Table 5 show that LDA prior parameters affect the quality of topic representation and the final Precision@200, but SES-HI does not work only under the default auto configuration. When $\lambda_{DT}$ is fixed to auto and $\lambda_{TW}$ is varied, Precision@200 remains between 21.00% and 22.60%, and the highest Precision@200 is obtained at $\lambda_{TW}=0.01$. This indicates that a moderately sparse topic-word prior can generate a clearer distribution of topic keywords, thereby benefiting the topic-guided expansion in Algorithm 4. When $\lambda_{TW}$ is fixed to 0.01 and $\lambda_{DT}$ is varied, most small values still maintain performance close to the baseline. However, when $\lambda_{DT}=0.05$, Precision@200 drops markedly to 17.20%, which suggests that an overly large document-topic prior makes document-topic distributions too smooth and weakens topic discrimination. When $\lambda_{TW}$ uses auto and $\lambda_{DT}$ takes fixed small values, the results are generally lower than the baseline. This indicates that, under the TREC experimental setting in this paper, automatically learning the document-topic prior together with a smaller topic-word prior is more suitable for obtaining stable topic representations.

Algorithm 4 indeed depends on the quality of the LDA topic model, but the added parameter sensitivity analysis shows that its performance variation is observable and explainable. The default configuration $\lambda_{DT}=\mathrm{auto}$ and $\lambda_{TW}=\mathrm{auto}$ is not the only usable setting, and $\lambda_{DT}=\mathrm{auto}$ with $\lambda_{TW}=0.01$ can achieve a higher Precision@200. Therefore, this paper supplements the analysis to more clearly explain the influence of LDA prior parameters on topic-guided expansion and the boundary of the proposed method.

### 8.4. Discussion

Integrating the theoretical analysis with the experimental results, we summarize the core strengths, performance, and application value of the proposed scheme, while also discussing its limitations and potential future research directions.

The proposed scheme demonstrates significant advantages in both retrieval efficiency and accuracy. Experimental evaluations reveal that during the index construction phase, the scheme exhibits a trend similar to EPSMR. Although the two-stage clustering process generates a larger number of nodes, leading to slightly higher construction time, it establishes a superior structural foundation for efficient retrieval. In the trapdoor generation phase, the introduction of semantic query expansion incurs higher overhead compared to EPSMR without expansion, but it effectively yields a more accurate semantic representation of the queries. During query execution, the scheme consistently outperforms EPSMR across various parameter settings due to the high semantic consistency afforded by the two-stage clustering, resulting in significantly improved pruning efficiency. Overall, the experimental results validate that the proposed scheme achieves a favorable balance between efficiency and retrieval quality. Compared with EPSMR, CASE-SSE, and SSS KG, SES-HI obtains more competitive retrieval precision and lower query latency under the tested TREC setting. PBINS benefits from stronger Transformer-style sentence embeddings and can achieve higher top-ranked retrieval quality in some cases, but it also introduces additional construction and query overhead due to private-bin organization and high-capacity semantic representation. Therefore, the proposed scheme should be understood as a lightweight and encryption-compatible semantic retrieval framework that provides a practical efficiency–precision trade-off, rather than as a universal replacement for all stronger plaintext semantic representations.

By achieving a more favorable balance between efficiency and precision, the proposed scheme is well-suited for large-scale encrypted document retrieval scenarios in cloud environments that demand high retrieval quality, such as digital libraries and enterprise knowledge bases. The hierarchical index structure and semantic enhancement strategies provide a viable pathway for high-quality semantic search in the ciphertext domain. However, the scheme is subject to certain limitations, which are explicitly discussed here to clarify the practical boundary of the proposed construction. First, the current security model follows the common honest-but-curious model in searchable encryption. Under this model, the cloud server is assumed to execute the protocol faithfully, while attempting to infer information from the encrypted index, trapdoors, access paths, returned identifiers, and score patterns. Therefore, threats involving malicious servers, such as result tampering, false-result injection, adaptive probing, inference attacks beyond the stated leakage profile, and requirements for verifiable result integrity, are beyond the threat model of this work. Moreover, the current construction assumes a single trusted data owner responsible for key generation and encrypted index construction. Multi-owner or multi-authority settings, including cross-owner key management, authorization, and index coordination, are beyond the scope of this work. Second, the correctness definition should be interpreted as algorithmic consistency between the encrypted search procedure and the corresponding plaintext search procedure under the same vector representation, index structure, pruning threshold, and top-*K* rule. It does not imply equivalence to an ideal semantic retrieval oracle independent of the adopted semantic representation. Third, the security, efficiency, and scalability of SES-HI depend on the underlying inner-product encryption primitive. Although the proposed hierarchical index, semantic query expansion, and pruning strategies are orthogonal to the specific primitive, different instantiations such as EASPE or IPE may lead to different leakage profiles and computational costs. Therefore, the reported performance should be interpreted as the performance of SES-HI under the selected inner-product encryption setting, while alternative primitives may change the absolute runtime, storage cost, and leakage characteristics without changing the high-level indexing and pruning design. Finally, since Algorithm 6 independently encrypts internal node vectors and document vectors, the resulting encrypted node vectors introduce additional storage overhead as the dataset grows. Consequently, the storage cost grows approximately with the number of encrypted document vectors and internal node summaries, which may become a practical concern for very large-scale deployments. This overhead is exchanged for more effective branch pruning and lower query latency, but more compact encrypted node representations and index compression remain important optimization directions.

The experimental evaluation in this work is conducted on the TREC document collection, which is a widely adopted benchmark dataset in information retrieval and searchable encryption research. The use of TREC enables fair comparison with existing methods because it provides standardized document collections, query sets, and relevance judgments. However, we acknowledge that evaluation on a single benchmark dataset cannot fully demonstrate the generalization capability of the proposed SES-HI framework under diverse semantic distributions and domain-specific document collections. Different datasets can exhibit distinct vocabulary characteristics, topic distributions, and document densities, which could influence clustering quality, query expansion effectiveness, and pruning behavior. Therefore, although the reported results demonstrate the effectiveness of SES-HI in a standard retrieval environment, additional validation on larger-scale, multi-domain, and heterogeneous datasets remains necessary. Extending the framework to broader document collections and investigating its robustness under varying semantic distributions will be an important direction for future research.

Future work can focus on several key areas: supporting dynamic updates, enhancing semantic representation, optimizing the encrypted index structure, strengthening security against stronger adversarial models, and extending support for diverse query types. Specifically, research efforts could investigate efficient index maintenance mechanisms that support document addition and deletion to accommodate dynamically changing data. Exploring semantic vector representations based on pre-trained language models (e.g., BERT) could further improve semantic understanding capabilities. In particular, integrating verifiable search mechanisms, ORAM-based access-pattern protection, differential privacy, or verifiable result integrity could further improve robustness against malicious servers, adaptive probing, and inference attacks. Meanwhile, compact node-vector encoding, shared encrypted representations, and index compression can help reduce the storage overhead caused by independently encrypted internal nodes and document vectors. To reduce overhead without compromising retrieval accuracy, future work may design more compact encrypted index structures or parallel retrieval strategies. Extending the scheme to support more complex query types is another valuable direction. Through these improvements, the scheme’s practicality and robustness in larger-scale, multi-source, and heterogeneous data environments can be further enhanced.

## 9. Conclusions

This paper presents a novel scheme (SES-HI) that supports efficient and accurate semantic retrieval over encrypted cloud data. At its core, the scheme introduces a hierarchical ciphertext index based on document topic vectors. Specifically, documents are first grouped into semantically coherent clusters through a two-stage clustering procedure employing Ward’s method and *k*-means. These clusters are subsequently organized into a balanced ω-ary search tree. The hierarchical index condenses the semantic information of each cluster or subtree into a representative node vector, which in turn facilitates effective pruning during encrypted search.

To enhance query semantics, we propose a screening-based query expansion mechanism that leverages both LDA and Word2Vec, effectively enriching short queries while controlling noise. The search process executes a depth-first traversal on the encrypted index, incorporating global and top-*K* competitive pruning strategies. The scheme is implemented using an encryption primitive that supports inner products, allowing the cloud server to perform relevance computations directly over ciphertexts.

Formal security analysis under a well-defined leakage profile confirms the scheme’s privacy guarantees in the honest-but-curious model. We also explicitly clarify that malicious servers, adaptive probing, result tampering, inference attacks beyond the stated leakage profile, and verifiable result integrity are not covered by the current threat model and should be addressed in future extensions. Extensive evaluations on the TREC dataset, including comparisons with EPSMR, CASE-SSE, PBINS, and SSS KG, demonstrate that SES-HI achieves a competitive retrieval precision while maintaining lower query latency than most semantic encrypted retrieval baselines. Although PBINS benefits from stronger Transformer-style sentence embeddings in some top-ranked retrieval metrics, SES-HI provides a more lightweight and encryption-compatible trade-off among semantic representation, index construction, trapdoor generation, and query execution. Moreover, since SES-HI is instantiated over an inner-product encryption primitive and encrypts node and document vectors separately, its concrete efficiency, leakage profile, and storage cost are influenced by the selected cryptographic primitive and index representation. This work thus provides a practical and effective solution for privacy-preserving semantic search in cloud environments.

## Figures and Tables

**Table 1:** Theoretical time complexity comparison of different schemes

Scheme	Index Construction	Trapdoor Generation	Query Execution
Proposed scheme	$O\;\left(\left[\frac{n(\omega +1)}{\gamma \omega }+n\right]t^{2}\right)$	$O(\rho \beta t+t^{2})$	$O\;\left(t\log_{\omega }\left(n/\gamma \right)+t\gamma \right)$
EPSMR	$O\;\left(\left[\frac{n(\omega +1)}{\gamma \omega }+n\right]t^{2}\right)$	$O(t^{2})$	$O\;\left(t\log_{\omega }\left(n/\gamma \right)+t\gamma \right)$
CASE-SSE	$O(km^{2})$	$O(\beta m+m^{2})$	O(mlogk)
PBINS	$O\;\left(\left(n+k\right)t^{2}\right)$	$O(t^{2})$	$O\;\left(\left(k+\frac{n}{k}\right)t\right)$
SSS KG	$O\;\left(\left(|V\right|+\left|E|\right)t^{2}\right)$	$O\;\left(\left(\right|V_{Q}\left|+\right|E_{Q}\left|\right)t^{2}\right)$	O(|DB[v]|t)

**Table 2:** Fine-grained retrieval quality under different document scales.

Dataset Size	Metric	Proposed Scheme	PBINS	EPSMR	SSS KG	CASE-SSE
1w	Precision@10	5.00%	14.00%	10.00%	4.00%	5.00%
	Precision@20	7.00%	9.50%	7.00%	4.50%	3.50%
	NDCG@10	21.53%	27.05%	25.06%	17.23%	21.53%
2w	Precision@10	5.00%	5.00%	3.00%	3.00%	1.50%
	Precision@20	5.00%	5.50%	4.75%	3.75%	1.00%
	NDCG@10	12.53%	12.53%	11.12%	11.12%	6.46%
3w	Precision@10	6.67%	6.00%	6.67%	6.67%	4.00%
	Precision@20	5.50%	5.00%	6.00%	4.33%	2.67%
	NDCG@10	9.81%	9.56%	9.81%	9.81%	8.68%
4w	Precision@10	6.25%	6.00%	6.50%	4.50%	1.75%
	Precision@20	5.88%	4.75%	5.63%	3.75%	1.88%
	NDCG@10	7.73%	7.65%	7.79%	7.17%	5.74%
5w	Precision@10	5.40%	6.00%	4.00%	3.40%	2.80%
	Precision@20	4.90%	5.60%	3.80%	2.80%	1.70%
	NDCG@10	6.28%	6.43%	5.88%	5.66%	5.41%

**Table 3:** Fine-grained retrieval quality under different semantic vector dimensions.

Dimension	Metric	Proposed Scheme	PBINS	EPSMR	SSS KG	CASE-SSE
120	Precision@10	3.80%	–	2.80%	–	2.80%
	Precision@20	4.10%	–	3.10%	–	1.70%
	NDCG@10	5.81%	–	5.41%	–	5.41%
140	Precision@10	2.80%	–	2.40%	–	1.60%
	Precision@20	2.30%	–	2.10%	–	1.00%
	NDCG@10	5.41%	–	5.21%	–	4.73%
160	Precision@10	5.40%	–	4.00%	–	2.20%
	Precision@20	4.90%	–	3.80%	–	1.20%
	NDCG@10	6.28%	–	5.88%	–	5.11%
180	Precision@10	6.20%	–	6.00%	–	2.40%
	Precision@20	4.90%	–	4.90%	–	1.40%
	NDCG@10	6.47%	–	6.43%	–	5.21%
200	Precision@10	4.00%	–	2.80%	3.40%	1.80%
	Precision@20	4.40%	–	3.00%	2.80%	1.10%
	NDCG@10	5.88%	–	5.41%	5.66%	4.87%
384	Precision@10	–	6.00%	–	–	–
	Precision@20	–	5.60%	–	–	–
	NDCG@10	–	6.43%	–	–	–

**Table 4:** Ablation results of the expansion similarity threshold δ. The meaning of the arrow is that compared with the baseline, the arrow pointing upwards indicates higher accuracy, while the arrow pointing downwards indicates lower accuracy.

*T*	*W*	δ	Precision@200
0	0	0	22.60% baseline
3	3	0	20.50% ↓
3	3	0.3	22.80% ↑
3	3	0.5	24.70% ↑
3	4	0	20.70% ↓
3	4	0.3	23.30% ↑
3	4	0.5	24.80% ↑
3	5	0	20.60% ↓
3	5	0.3	24.20% ↑
3	5	0.5	25.50% ↑
4	4	0.35	23.90% ↑
4	4	0.4	24.10% ↑
4	4	0.45	24.50% ↑
4	4	0.5	26.70% ↑
4	4	0.55	25.80% ↑
4	5	0.5	25.50% ↑
5	5	0.3	23.00% ↑
5	5	0.5	25.80% ↑
5	5	0.6	25.10% ↑

**Table 5:** Sensitivity analysis of LDA prior parameters. The meaning of the arrow is that compared with the baseline, the arrow pointing upwards indicates higher accuracy, while the arrow pointing downwards indicates lower accuracy.

$\lambda_{\mathrm{DT}}$	$\lambda_{\mathrm{TW}}$	Precision@200	Change
auto	auto	21.10%	baseline
auto	0.001	21.00%	↓−0.10 pp
auto	0.005	22.00%	↑ +0.90 pp
auto	0.008	21.00%	↓−0.10 pp
auto	0.01	22.60%	↑ +1.50 pp
auto	0.012	22.30%	↑ +1.20 pp
auto	0.02	21.00%	↓−0.10 pp
0.001	0.01	21.20%	↑ +0.10 pp
0.005	0.01	21.50%	↑ +0.40 pp
0.008	0.01	21.30%	↑ +0.20 pp
0.01	0.01	21.20%	↑ +0.10 pp
0.012	0.01	21.40%	↑ +0.30 pp
0.05	0.01	17.20%	↓−3.90 pp
0.001	auto	18.50%	↓−2.60 pp
0.005	auto	18.90%	↓−2.20 pp
0.01	auto	19.60%	↓−1.50 pp
0.012	auto	19.30%	↓−1.80 pp
0.05	auto	18.20%	↓−2.90 pp

## Data Availability

The data supporting the reported results were derived from the standard TREC test collections. The TREC collections include standard query topics and relevance judgment files, qrels, which were used for retrieval evaluation in this study. Access to the dataset is managed by the Text REtrieval Conference, TREC, and users should apply for the dataset through the official TREC website, https://trec.nist.gov/, in accordance with its data access and usage policies.

## Abbreviations

The following abbreviations are used in this manuscript:

Acronym	Full Name
SE	Searchable Encryption
SSE	Symmetric Searchable Encryption
SES-HI	Semantically Enhanced Searchable Encryption with Hierarchical Index
DO	Data Owner
DU	Data User
CS	Cloud Server
LDA	Latent Dirichlet Allocation
Word2Vec	Word-to-Vector Model
EASPE	Enhanced Asymmetric Scalar Product Encryption
IPE	Inner Product Encryption
TREC	Text REtrieval Conference
EPSMR	Efficient Privacy-Preserving Semantic-Aware Multi-Keyword Ranked Search
CASE-SSE	Context-Aware Semantically Extensible Searchable Symmetric Encryption
PBINS	Private Bin-based Searchable Encryption
SSS KG	Semantic Searchable Scheme based on Knowledge Graph
DPMRS	Dictionary Partition-Based Multi-Keyword Ranked Search Scheme
DPVSM	Dictionary Partition-Based Vector Space Model
DPKD-index	Dictionary Partition-Based Keyword Distribution Inverted Index
DSTree-index	Double-Tier Search Tree-Based Index
IND2-CKA	Indistinguishability under Adaptive Chosen-Keyword Attack
ORAM	Oblivious RAM
TF-IDF	Term Frequency-Inverse Document Frequency
SBERT	Sentence-BERT
NDCG	Normalized Discounted Cumulative Gain
Top-*K*	Top-*K* Ranked Results
